# Automated Echocardiographic Detection of Heart Failure With Preserved Ejection Fraction Using Artificial Intelligence

**DOI:** 10.1016/j.jacadv.2023.100452

**Published:** 2023-07-28

**Authors:** Ashley P. Akerman, Mihaela Porumb, Christopher G. Scott, Arian Beqiri, Agisilaos Chartsias, Alexander J. Ryu, William Hawkes, Geoffrey D. Huntley, Ayana Z. Arystan, Garvan C. Kane, Sorin V. Pislaru, Francisco Lopez-Jimenez, Alberto Gomez, Rizwan Sarwar, Jamie O'Driscoll, Paul Leeson, Ross Upton, Gary Woodward, Patricia A. Pellikka

**Affiliations:** a Ultromics Ltd, Oxford, United Kingdom; b Department of Quantitative Health Sciences, Mayo Clinic, Rochester, Minnesota, USA; c Division of Hospital Internal Medicine, Mayo Clinic, Rochester, Minnesota, USA; d Department of Cardiovascular Medicine, Mayo Clinic, Rochester, Minnesota, USA; e Cardiovascular Clinical Research Facility, University of Oxford, Oxford, United Kingdom; f Experimental Therapeutics, Medical Sciences Division, Radcliffe Department of Medicine, University of Oxford, Oxford, United Kingdom; g Department of Cardiology, St George’s University Hospitals NHS Foundation Trust, London, United Kingdom

**Keywords:** diastolic function, echocardiography, heart failure, imaging, machine learning

## Abstract

**Background:**

Detection of heart failure with preserved ejection fraction (HFpEF) involves integration of multiple imaging and clinical features which are often discordant or indeterminate.

**Objectives:**

The authors applied artificial intelligence (AI) to analyze a single apical 4-chamber transthoracic echocardiogram video clip to detect HFpEF.

**Methods:**

A 3-dimensional convolutional neural network was developed and trained on apical 4-chamber video clips to classify patients with HFpEF (diagnosis of heart failure, ejection fraction ≥50%, and echocardiographic evidence of increased filling pressure; cases) vs without HFpEF (ejection fraction ≥50%, no diagnosis of heart failure, normal filling pressure; controls). Model outputs were classified as HFpEF, no HFpEF, or nondiagnostic (high uncertainty). Performance was assessed in an independent multisite data set and compared to previously validated clinical scores.

**Results:**

Training and validation included 2,971 cases and 3,785 controls (validation holdout, 16.8% patients), and demonstrated excellent discrimination (area under receiver-operating characteristic curve: 0.97 [95% CI: 0.96-0.97] and 0.95 [95% CI: 0.93-0.96] in training and validation, respectively). In independent testing (646 cases, 638 controls), 94 (7.3%) were nondiagnostic; sensitivity (87.8%; 95% CI: 84.5%-90.9%) and specificity (81.9%; 95% CI: 78.2%-85.6%) were maintained in clinically relevant subgroups, with high repeatability and reproducibility. Of 701 and 776 indeterminate outputs from the Heart Failure Association-Pretest Assessment, Echocardiographic and Natriuretic Peptide Score, Functional Testing (HFA-PEFF), and Final Etiology and Heavy, Hypertensive, Atrial Fibrillation, Pulmonary Hypertension, Elder, and Filling Pressure (H2FPEF) scores, the AI HFpEF model correctly reclassified 73.5% and 73.6%, respectively. During follow-up (median: 2.3 [IQR: 0.5-5.6] years), 444 (34.6%) patients died; mortality was higher in patients classified as HFpEF by AI (HR: 1.9 [95% CI: 1.5-2.4]).

**Conclusions:**

An AI HFpEF model based on a single, routinely acquired echocardiographic video demonstrated excellent discrimination of patients with vs without HFpEF, more often than clinical scores, and identified patients with higher mortality.

Heart Failure (HF) is a clinical syndrome affecting over 64 million people worldwide and has an increasing prevalence.^1^^,^^2^ Measurement of ejection fraction (EF) is used to categorize HF; while HF with reduced EF is relatively simple to identify, heart failure with preserved ejection fraction (HFpEF) is more complex, leading to differences in diagnostic criteria,^3^ and likely contributing to “failed” clinical trials.^4^ However, with mounting evidence indicating a beneficial impact of sodium-glucose cotransporter-2 inhibitors across the spectrum of HF,^5^ a key focus must now be improving diagnostic capacity^6^ in a patient population with poor 5-year survival rates, high hospital readmission rates, and substantial morbidity.^7^^,^^8^

HFpEF is a heterogenous syndrome associated with various comorbidities, wherein cardiac and noncardiac factors contribute to elevated intracardiac filling pressure, resulting in signs and symptoms of HF.^3^^,^^9^ Although transthoracic echocardiography (TTE) is routinely used to estimate intracardiac filling pressure,^9^^,^^10^ there is considerable variability in its performance and interpretation, and a high burden on skills, time, and expertise for acquiring diagnostic quality information which may not be feasible beyond expert clinical sites. Clinical algorithms, utilizing multiple sources of patient data,^11^^,^^12^ may be limited by discordant or incomplete data.^13^^,^^14^ These factors collectively contribute to variable diagnostic capacity, increasing the requirement for invasive confirmatory tests (eg, right heart catheterization^9^^,^^12^), adding further burden to the patient and health care system, and potentially missing individuals who might benefit from treatment.

Recent work in artificial intelligence (AI) computer vision techniques offer great promise that computational methods can better interpret the vast amount of information that exists within medical data including images. Whereas recent AI studies have combined clinical parameters and manual echocardiographic measurements to classify diastolic dysfunction and HFpEF,^15^, ^16^, ^17^ fewer have used echocardiographic images.^18^^,^^19^ Development of an approach using this simple input might obviate the need for complex Doppler assessment, provide supporting information when traditional measures are nondiagnostic, or limit data requirements when such data collection is not feasible.

The objective of this study was to develop an AI model to automatically detect HFpEF by only using the apical 4-chamber (A4C) TTE video clip. This view was selected because it includes much information (chamber sizes, wall thicknesses, annulus motion, etc) and is routinely acquired in imaging protocols. In an independent data set, we tested the hypothesis that the developed AI HFpEF model would demonstrate acceptable classification accuracy, and feasibility superior to current clinical scores for detection of HFpEF.

## Methods

### Data sources and study population

This retrospective, multisite, and multinational cohort study was approved by Institutional Review Boards of Mayo Clinic, United States and St. George’s University Hospitals, National Health Service Foundation Trust, United Kingdom. Patients provided written informed consent for inclusion in research; consent for use of TTE analysis and relevant clinical patient information was exempted by the participating Institutional Review Boards due to the use of deidentified data. Data from the United States and United Kingdom were used in the training and validation of the AI model, whereas independent multisite data from the United States were used for testing.

#### Model training and validation

**Figure 1:**
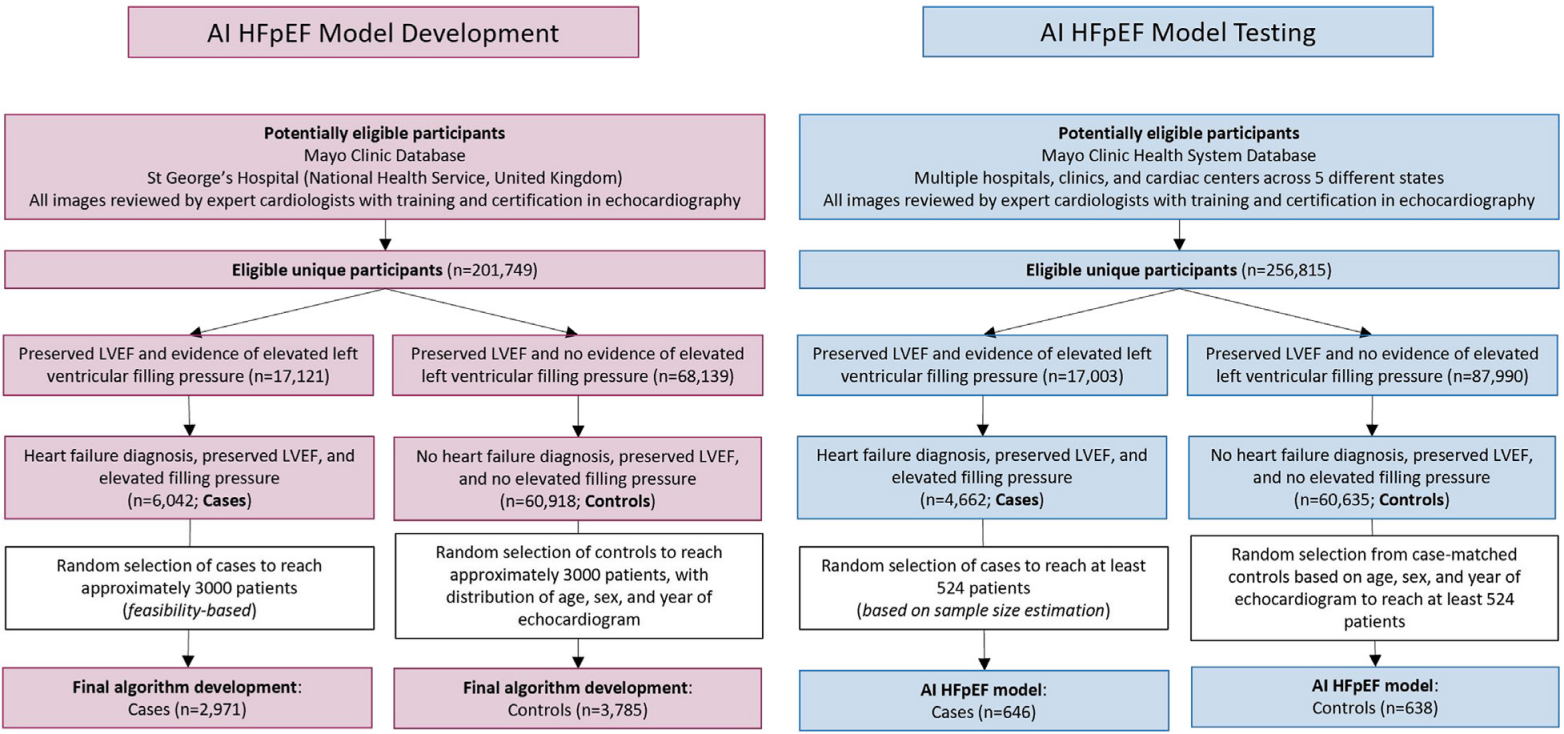
**Flow Diagram Illustrating Identification and Selection of Patients in AI HFpEF Model Development and Testing** AI = artificial intelligence; HFpEF = heart failure with preserved ejection fraction; LVEF = left ventricular ejection fraction.

The Mayo Clinic echocardiography database, which comprises all clinical images and TTE reports since 2002, and matched electronic medical records were screened for patients meeting the ground truth determination for cases and controls. Data were included for patients who had undergone a comprehensive TTE at Mayo Clinic in Rochester, Minnesota between January 2009 and December 2020. Echocardiograms at Mayo Clinic are performed by certified cardiac sonographers and interpreted by experienced level 3 trained physicians prior to the patient’s dismissal from the laboratory. A continuous random sampling of the data pool was taken and cross-referenced for preserved EF, and evidence of increased intracardiac filling pressure, until the desired number of cases was compiled (Figure 1). Controls were then randomly sampled to achieve a distribution of age, sex, and year of echocardiogram amongst patients. St. George’s Hospital cardiac database was screened in an identical manner to the Mayo Clinic echocardiography database to enrich the data set and facilitate generalizability via multinational data.

#### Independent testing of the AI HFpEF model

Multicenter independent retrospective data were collected within Mayo Clinic Health System to test the AI HFpEF model. Patients were selected from geographically distinct areas from the data used in model development to ensure generalizability. Data were selected from clinical sites spanning 4 states, and outreach services across 5 states (Supplemental Table 1). Cases and controls were matched for sex and year of echocardiogram and attempts were made to match for age. To better assess generalizability, up-sampling of non-White and Hispanic populations was used.

### Identification of study groups

The ground truth determination used in model training, validation and independent testing was based on data collected from patient medical records and comprehensive TTE reports. The definition of cases was consistent with the current national guidelines for detection and diagnosis of HF,^9^ based on the clinical diagnosis provided by the treating physician, and matching the clinical patient pathway for this patient cohort. Patients with HFpEF (cases) and patients without HFpEF (controls) were therefore identified via the mechanisms described below and illustrated in Figure 1.

#### Clinical diagnosis of heart failure

Documented clinical diagnosis of HF, based on an International Classification of Diseases 9 or 10 code, within 1 year of the associated echocardiogram (case) or lack of this diagnosis (control) was collected from the patient medical records (Supplemental Table 2).

#### Preserved systolic function

Documented evidence of preserved systolic function according to TTE (cases and controls) was obtained from the patient TTE reports. This was evidenced by a left ventricular EF of at least 50%^20^ (Supplemental Appendix), obtained using standard echocardiographic procedures at the relevant site, and interpreted by qualified clinicians.

#### Evidence of elevated intracardiac filling pressure

Documented evidence of increased intra-cardiac filling pressure (cases) or lack thereof (controls), was obtained from comprehensive clinical TTE reports, measured in accordance with relevant guidelines^9^^,^^10^ (Supplemental Appendix, Figure 1).

### Overview of the AI HFpEF model

Model training and validation were completed using Python (version 3.7.7) with TensorFlow (version 2.2) on a rack-mounted server with a set of 3 Nvidia Tesla V100 graphic processing units, each with 32 GB of video RAM. Model inputs consisted of only A4C TTE video clips. For training and validation of the AI HFpEF model, all A4C video clips for a given patient were used.

A convolutional neural network (CNN)^21^ model was applied to the A4C video clips. The model was comprised of 3 series of 3-dimensional (3D) convolutional layers. Each of these 3 series was a sequence of 2 convolutions with a 3 × 3 × 3 kernel, followed by batch normalization and rectified linear unit activation, and then 1 max-pooling operation with kernel size and stride of 3 in every direction. This architecture was chosen since it is well suited to operate on 3-dimensional data (2 in plane spatial dimensions for each frame plus time). The input of the model was comprised of all overlapping sequences of 30 frames, with a stride of 10 frames, from the entire A4C video clip which was usually comprised of 3 cardiac cycles. The fully connected layer used a dropout with a 0.5 probability (Central Illustration).

**Central Illustration:**
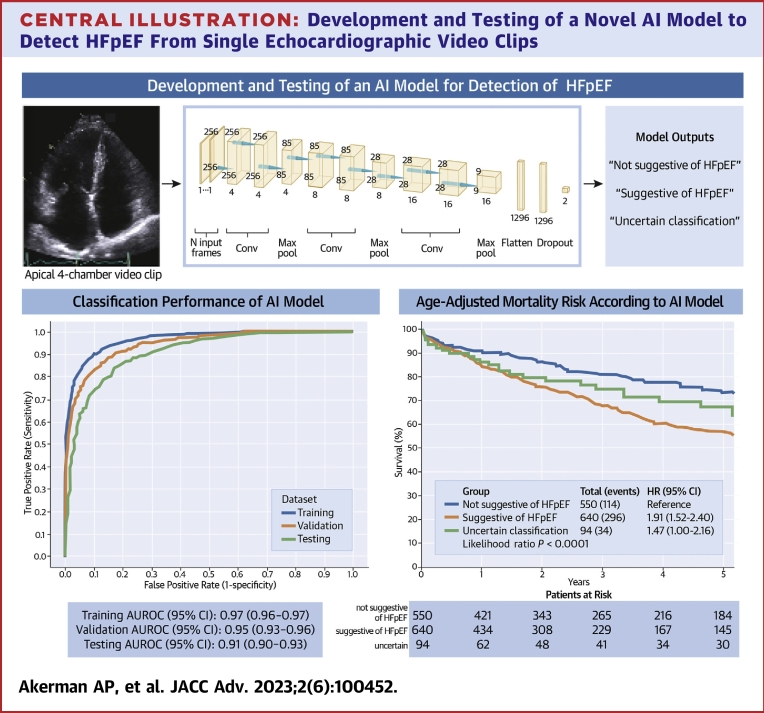
**Development and Testing of a Novel AI Model to Detect HFpEF From Single Echocardiographic Video Clips** A 3-dimensional convolutional neural network was developed **(middle)** to detect heart failure with preserved ejection fraction using only apical 4-chamber video clips. Discrimination performance was excellent (area under receiver-operating characteristic curve; **bottom left**), and age-adjusted risk of mortality was higher when patients received from the model a diagnostic output suggestive of heart failure with preserved ejection fraction compared to a diagnostic output not suggestive of heart failure with preserved ejection fraction **(bottom right)**. AI = artificial intelligence; AUROC = area under receiver-operating characteristic curve; HFpEF = heart failure with preserved ejection fraction.

All A4C video clips were subjected to automated image preprocessing prior to being fed into the neural network, which included extraction of the Digital Imaging and Communications in Medicine ultrasound region, resizing each frame to 256 × 256 pixels, and frame-wise normalization. The CNN input data were stored as Python NumPy arrays and fed into the model 30 consecutive frames at a time. The final prediction score was computed as the mean of the prediction probabilities obtained when evaluating all consecutive sequences of 30 frames in a video clip that overlapped with a stride of 1 frame.

Data augmentation was applied randomly throughout training to improve the generalizability of the model; augmentations included horizontal flipping, central cropping, random rotations, and random brightness. The 3D CNN model was trained using a cross-entropy loss function and Adam optimizer with an initial learning rate of 3 × 10^−4^ and a batch size of 64. The learning rate was reduced by a factor of 0.9 when the validation loss stopped improving for 20 optimization steps. Total training time was approximately 8 hours using a single Tesla V100 graphic processing unit.

#### AI HFpEF model outputs

The AI HFpEF model used a softmax activation function in its final layer to calculate a value between 0 and 1, which was mapped to a binary negative and positive diagnostic prediction of HFpEF, respectively. The classification threshold for the output predictions on the validation data set was set to 0.5, computed on all points on the receiver operator characteristic curve.

Finally, a nondiagnostic output was generated based on model uncertainty, using the expected entropy on all predictions across the consecutive sequences of 30 frames. The threshold for expected entropy (0.59) was determined according to the threshold at which classification performance was improved significantly without omitting more than 10% of the data during model training (Supplemental Figure 1).

### Comparison of AI model with current clinical practice

To test the hypothesis that the classification accuracy of the developed AI HFpEF model, based on analysis of a single A4C video clip, was acceptable, we compared observed sensitivity and specificity in the independent testing data set to average reported data in the literature (sensitivity, 74%; specificity, 65%) (Supplemental Appendix). To demonstrate a 5% increase from these benchmarks, and allowing for 21.9% of nondiagnostic outcomes, ∼1,048 patients were required in the independent testing data set (Supplemental Appendix). Classification performance was assessed in a priori determined subgroups of interest related to patient demographics, clinical, and echocardiographic criteria (Supplemental Appendix). The previously validated clinical Heart Failure Association-Pretest Assessment, Echocardiographic and Natriuretic Peptide Score, Functional Testing, and Final Etiology (HFA-PEFF)^12^ and Heavy, Hypertensive, Atrial Fibrillation, Pulmonary Hypertension, Elder, and Filling Pressure (H2FPEF) scores^11^ were calculated retrospectively (ie, they were not required for the original clinical diagnosis) and categorized as unlikely (0 or 1), indeterminate (2-4), or probable (5-6) likelihood of HFpEF for the HFA-PEFF score, and low probability (0 or 1), indeterminate (2-5), or high probability (6-9) of HFpEF for the H2FPEF score. The impact of incorporating the AI HFpEF model into current clinical practice was assessed using decision curve analysis (Supplemental Appendix).

### Statistical analysis

Statistical optimization of the CNN was completed as described above. Measures of model calibration (Hosmer-Lemeshow goodness-of-fit test) (Supplemental Appendix) and classification performance (area under receiver-operating characteristic curve [AUROC], sensitivity, and specificity) were assessed during development and for the final model. Gradient-weighted class activation mapping (Grad-CAM^22^) method was employed for visualizing the most important regions in the input images for the model to discriminate between case and control.

Mortality was evaluated using the Kaplan-Meier method, censoring subjects at last known follow-up. The survival curve was adjusted for age using a direct adjustment method based on averaging the Cox model derived survival curves of each patient. Cox proportional hazards regression was also used to estimate the HR of mortality between groups based on model outputs with adjustments for age. Repeatability and reproducibility were assessed on classification predictions on all amenable images collected for the primary objective, including images which were nondiagnostic due to high uncertainty. For repeatability, the same image clip was read twice by the device. For reproducibility, patients from the main testing data set with 2 image clips were extracted and used for analysis.

Data are reported as mean ± SD [sample size], and where appropriate, the *t*-test, analysis of variance, Chi-squared test, or Wilcoxon rank sum test were used to examine differences between groups. 95% CIs for AUROC were calculated using the DeLong method. All other inferential statistics are reported as point estimates and associated 95% CIs (lower bound-upper bound), calculated using bootstrap methods. Unless otherwise stated, statistical tests were 2-sided, with alpha <0.05 considered statistically significant. Analyses were performed using R (version 4.1) and Python (version 3.7.7).

## Results

### AI HFpEF model development

From an available 6,823 patients (3,004 cases, 3,819 controls), and 7,321 video clips (3,217 cases, 4,104 controls), 7 video clips could not be read, and 65 contained <30 frames required for the analysis. St George’s (United Kingdom) data contributed ∼3% to the total training and validation data set with cases (n = 140) and controls (n = 92). Thus, the final model training and validation data set comprised 6,756 patients (2,971 cases, 3,785 controls) with 7249 A4C video clips (3,185 cases, and 4,064 controls; 16.8% of patients retained for validation holdout) (Figure 1, Table 1).

**Table 1:** Characteristics for Patients With and Without HFpEF Used in Model Training, Validation, and Independent Testing

	Controls (Training)	Controls (Validation)	Controls (Testing)	Cases (Training)	Cases (Validation)	Cases (Testing)
Patient demographics						
Age, y	55.8 ± 15.7 [3,047]	57.5 ± 15.8 [644]	64.6 ± 17.4 [638]	73.2 ± 11.5 [2,420]	73.7 ± 11.5 [411]	72.4 ± 13.3 [646]
Women	1,632 (52.2)	344 (52.4)	326 (51.1)	1,277 (50.5)	237 (53.7)	337 (52.2)
BMI, kg/m^2^	28.1 ± 6.4 [3,043]	28.4 ± 6.4 [643]	28.7 ± 6.8 [637]	30.8 ± 7.3 [2,416]	30.7 ± 7.0 [411]	30.6 ± 7.0 [646]
SBP, mm Hg	122 ± 18 [3,025]	123 ± 17 [644]	129 ± 20 [617]	132 ± 21 [2,402]	133 ± 21 [407]	139 ± 24 [635]
African American	93 (3.0)	21 (3.2)	127 (19.9)	48 (1.9)	6 (1.4)	124 (19.2)
White, non-Hispanic	2,817 (90.0)	602 (91.8)	383 (60.0)	2,253 (89.1)	385 (87.3)	399 (61.8)
Other	54 (1.7)	5 (0.8)	128 (20.1)	44 (1.7)	8 (1.8)	123 (19.0)
Comorbidities and risk factors						
Obesity	1,943 (62.1)	427 (65.1)	451 (70.7)	1,924 (76.1)	332 (75.3)	504 (78.0)
Hypertension	1,120 (35.8)	272 (41.5)	301 (47.2)	1,994 (78.8)	349 (79.1)	559 (86.5)
Hyperlipidemia	1,303 (41.6)	327 (49.9)	331 (51.9)	1,787 (70.6)	304 (68.9)	467 (72.3)
Structural heart disease	1,058 (33.8)	231 (35.2)	375 (58.8)	1,913 (75.6)	341 (77.3)	567 (87.8)
Atrial fibrillation	390 (12.5)	102 (15.6)	90 (14.1)	885 (35.0)	156 (35.37)	227 (35.1)
Coronary artery disease	248 (7.9)	48 (7.3)	65 (10.2)	849 (33.6)	137 (31.1)	204 (31.6)
Chronic kidney disease	97 (3.1)	27 (4.1)	75 (11.8)	624 (24.7)	109 (24.7)	302 (46.8)
Diabetes mellitus	364 (11.6)	92 (14.0)	107 (16.8)	1,003 (39.6)	171 (38.8)	326 (50.5)
Pulmonary disease	508 (16.2)	118 (18.0)	109 (17.1)	898 (35.5)	149 (33.8)	255 (39.5)
Previous cardiovascular or cerebrovascular event	282 (9.0)	70 (10.7)	116 (18.2)	933 (36.9)	161 (36.5)	264 (40.9)
Cardiovascular measurements						
LV mass index,^a^ g/m^2^	84 ± 17 [2,940]	83 ± 17 [618]	85 ± 21 [626]	112 ± 32 [2,255]	111 ± 29 [381]	121 ± 36 [630]
Relative wall thickness (ratio)	0.39 ± 0.06 [2,945]	0.39 ± 0.06 [619]	0.44 ± 0.08 [626]	0.44 ± 0.10 [2,260]	0.44 ± 0.09 [381]	0.5 ± 0.12 [631]
LA volume index,^a^ mL/m^2^	28.0 ± 7.5 [1844]	27.5 ± 7.2 [397]	28.8 ± 35.4 [237]	43.5 ± 12.0 [1,596]	43.9 ± 12.1 [283]	44.8 ± 15.2 [220]
Ejection fraction, %	63 ± 5 [3,047]	63 ± 5 [644]	63 ± 6 [638]	62 ± 6 [2,419]	62 ± 6 [411]	61 ± 6 [646]
Global longitudinal strain, %	−20 ± 3 [309]	−21 ± 2 [64]	−20 ± 2 [40]	−14 ± 4 [193]	−16 ± 3 [28]	−14 ± 4 [61]
Early diastolic mitral filling velocity (E-wave; cm/s)	73 ± 16 [3,029]	73 ± 16 [641]	73 ± 17.0 [634]	97 ± 23 [2,416]	99 ± 24 [410]	102 ± 26 [643]
E: A ratio	1.24 ± 0.46 [2,956]	1.22 ± 0.47 [627]	1.08 ± 0.4 [629]	1.45 ± 0.84 [2,399]	1.46 ± 0.75 [405]	1.52 ± 0.87 [635]
E-wave deceleration time, ms	198 ± 38 [2,871]	200 ± 39 [611]	212 ± 49 [612]	201 ± 50 [2,345]	195 ± 51 [386]	207 ± 62 [629]
Septal mitral annular early diastolic tissue velocity (e’; cm/s)	9.2 ± 3.3 [3,025]	8.9 ± 2.3 [642]	7.8 ± 2.3 [625]	5.2 ± 1.4 [2,389]	5.3 ± 1.5 [405]	5.0 ± 1.6 [626]
Septal E/e’ ratio	8.3 ± 2.0 [3,014]	8.5 ± 2.2 [640]	9.9 ± 2.9 [625]	19.9 ± 7.1 [2,388]	20.0 ± 7.0 [405]	21.5 ± 7.6 [626]
Lateral mitral annular early diastolic tissue velocity (e’; cm/s)	11.3 ± 3.8 [2,949]	11.1 ± 3.2 [616]	10.3 ± 3.8 [359]	7.1 ± 2.4 [2,159]	7.1 ± 2.1 [358]	6.7 ± 2.3 [403]
Lateral E/e’ ratio	6.9 ± 2.1 [2,939]	7.0 ± 2.1 [614]	7.8 ± 2.7 [359]	15.0 ± 6.4 [2,158]	15.1 ± 5.9 [358]	16.4 ± 6.3 [403]
Average E/e’ ratio	7.6 ± 1.9 [3,023]	7.8 ± 2.1 [641]	9.4 ± 2.7 [627]	17.7 ± 6.3 [2,400]	18.0 ± 6.3 [407]	19.9 ± 6.8 [632]
Pulmonary artery systolic pressure, mm Hg	29 ± 7 [2,470]	29 ± 8 [529]	30 ± 7 [431]	42 ± 14 [2,208]	42 ± 13 [378]	45 ± 14 [527]
Tricuspid regurgitation velocity, m/s	2.4 ± 0.3 [2,473]	2.4 ± 0.3 [530]	2.4 ± 0.3 [434]	2.9 ± 0.5 [2,213]	2.9 ± 0.5 [378]	3.0 ± 0.5 [531]
Biomarkers						
BNP, pg/mL	105 ± 143 [11]	74 ± 67 [3]	110 ± 132 [43]	604 ± 663 [128]	1,285 ± 3,269 [29]	668 ± 1,517 [104]
NT-proBNP, pg/mL	756 ± 3,822 [239]	291 ± 572 [54]	362 ± 538 [70]	3,257 ± 6,030 [1,462]	3,399 ± 6,215 [242]	6,152 ± 1,052 [267]
Clinical algorithms						
H2FPEF score (continuous)	33 ± 27 [3,129]	37 ± 28 [656]	42 ± 28 [638]	79 ± 26 [2,530]	77 ± 29 [441]	80 ± 23 [646]
H2FPEF: low	1,382 (44.2)	244 (37.2)	168 (26.3)	141 (5.6)	37 (8.4)	5 (0.8)
H2FPEF: high	165 (5.3)	45 (6.9)	59 (9.4)	1,134 (44.8)	202 (45.8)	276 (42.7)
H2FPEF: indeterminate	1,582 (50.6)	367 (56.0)	411 (64.4)	1,255 (49.6)	202 (45.8)	365 (56.5)
HFA-PEFF: unlikely	1787 (57.1)	358 (54.6)	333 (52.2)	143 (5.7)	34 (7.7)	12 (1.9)
HFA-PEFF: probable	10 (0.3)	0 (0)	10 (1.6)	971 (38.4)	180 (40.8)	228 (35.3)
HFA-PEFF: indeterminate	1,332 (42.6)	298 (45.4)	295 (46.2)	1,416 (56.0)	227 (51.5)	406 (62.9)

Values are mean ± SD [N] or n (%) [N].

BMI = body mass index; BNP = brain natriuretic peptide; H2FPEF = Heavy, Hypertensive, Atrial Fibrillation, Pulmonary Hypertension, Elder, and Filling Pressure; HFA-PEFF = Heart Failure Association-Pretest Assessment, Echocardiographic and Natriuretic Peptide Score, Functional Testing, and Final Etiology; HFpEF = heart failure with preserved ejection fraction; LA = left atrial; LAVi = left atrial volume index; LV = left ventricle; LVMi = left ventricular mass index; NT-proBNP = N-terminal pro brain natriuretic peptide; SBP = systolic blood pressure.

a Indexing was performed to body surface area. Average filling refers to the calculated mean of the septal and lateral mitral annular early diastolic tissue velocity when both metrics are available, or the available metric when only 1 is available. Categories within the “Comorbidities and risk factors” section only refer to individuals with the given condition present. Obesity refers to a BMI >25.0 kg/m^2^. Structural heart disease refers to the presence of an enlarged LA volume index (≥34 mL/m^2^) or LV mass index (≥116/96 g/m^2^ for males and females, respectively), a relative wall thickening >0.42, or a posterior wall thickness ≥12 mm. Pulmonary disease refers to the presence of lung disease or chronic obstructive pulmonary disorder. Previous cardio- or cerebrovascular event refers to the presence of a previous stroke, transient ischemic attack, coronary artery revascularization, or myocardial infarction. Pulmonary artery systolic pressure calculated as: 4 · (tricuspid regurgitation velocity)^2^ + estimated right atrial pressure (5 mm Hg). HFA-PEFF probability categories calculated according to Pieske et al^12^ Patients with a score of 0 or 1, between 2 and 4, and 5 or more, were denoted as unlikely, indeterminate, and probable likelihood of HFpEF, respectively. H2FPEF continuous and categorical scores were calculated according to Reddy et al.^11^ For the categorical score, patients with a score of 0 or 1, 2 to 5, or 6 to 9, were denoted as low, indeterminate, and high probability of HFpEF, respectively.

Classification performance in the training and validation data sets was high (AUROC: 0.97 [95% CI: 0.96-0.97] and 0.95 [95% CI: 0.93-0.96, respectively) (Central Illustration). At a threshold of 0.50, this corresponded to a sensitivity and specificity of 88.7% and 85.4%, respectively, in the validation data set.

**Figure 2:**
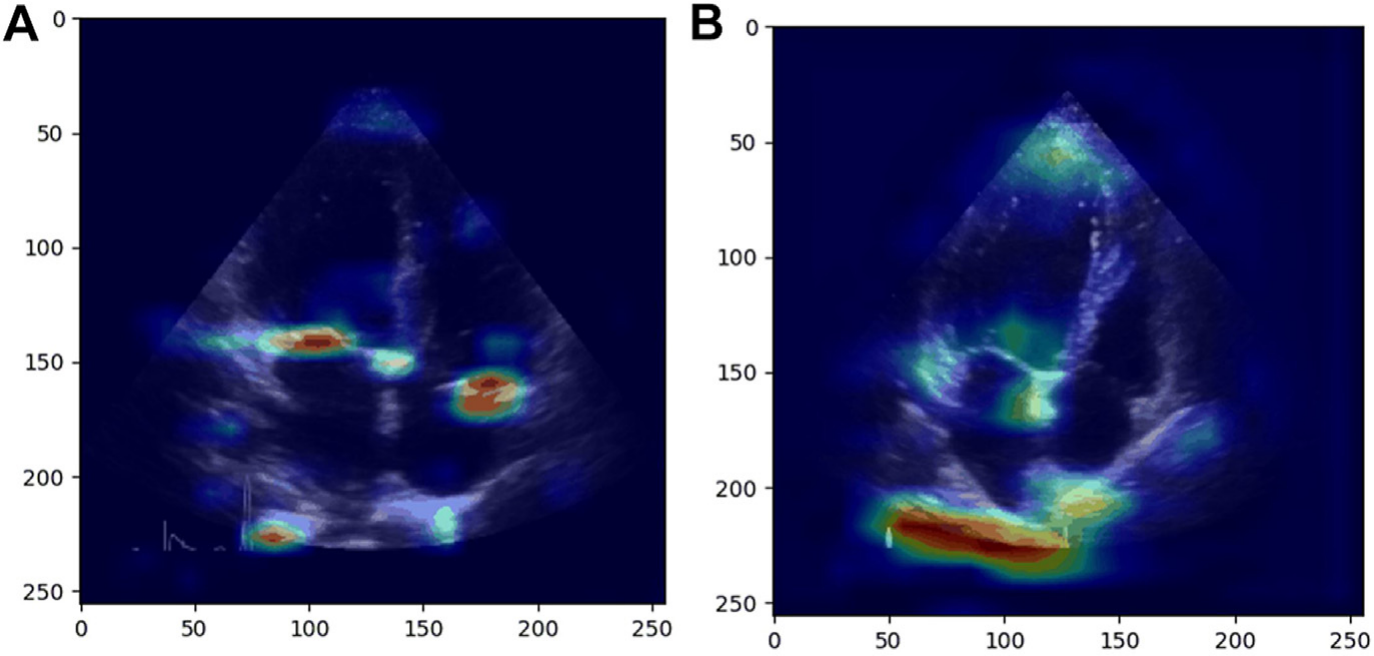
Grad-CAM Grad-CAM for correctly **(A)** and incorrectly classified **(B)** patient. Grad-CAM = gradient-weighted class activation mapping.

Figure 2 demonstrates representative Grad-CAM images for a correctly classified case, and an incorrectly classified control. The highlighted areas in the Grad-CAM identify “important” regions in the image to differentiate between cases and controls (Supplemental Appendix). In the correct example, the highlighted regions correspond to clearly defined cardiac structures with clinical importance, which suggesting that the model is “looking” at appropriate features. In the incorrect example, the strongest (red) signal appears in a less clearly defined structure/regions.

### Independent AI HFpEF model testing

In the independent testing data set, from an available 1,292 patients (650 cases and 642 controls), and 1,426 video clips (722 cases, 704 controls), 3 video clips could not be read, and 29 contained fewer than 30 frames required for the analysis. The final sample size for the independent testing data set was therefore 1,284 patients (646 cases, 638 controls) (Table 1).

#### Classification accuracy

The AI HFpEF model classified 94 out of 1,284 studies (7.3%) as non-diagnostic due to high model uncertainty. In the remaining data, sensitivity (87.8%; 95% CI: 84.5%-90.9%) and specificity (81.9%; 95% CI: 78.2%-85.6%) both exceeded the a priori benchmarks consistent with average clinical practice (both *P* < 0.001 for 1-sided Binomial Exact test), with corresponding positive and negative predictive values of 83.6% (95% CI: 80.2%-87.0%) and 86.5% (95% CI: 83.0%-90.0%), respectively. Compared to their correctly classified counterparts, misclassified controls were older with more evidence of structural heart disease and diastolic dysfunction, whereas the opposite was true for misclassified cases (Table 2).

**Table 2:** Summary of Characteristics for Patients With and Without HFpEF (Cases and Controls, Respectively) Who Were Correctly and Incorrectly Classified Using the AI HFpEF Model or Received No Classification due to High Model Uncertainty

	Controls (Correct)	Controls (Incorrect)	Controls (No Class)	*P* Value	Cases (Correct)	Cases (Incorrect)	Cases (No Class)	*P* Value
Patient demographics								
Age, y	61.4 ± 16.9 [476]	75.7 ± 14.3 [105]	71.1 ± 15.9 [57]	0.088	73.1 ± 12.9 [535]	68.2 ± 15.0 [74]	70.7 ± 14.7 [37]	0.351
Women	241 (50.6)	52 (49.5)	33 (57.9)	0.549	279 (52.2)	37.0 (50.0)	21.0 (56.8)	0.798
BMI, kg/m	28.4 ± 6.7 [476]	29.2 ± 6.3 [104]	30.3 ± 8.1 [57]	0.344	30.7 ± 7.0 [535]	29.4 ± 7.3 [74]	30.9 ± 6.2 [37]	0.260
SBP, mm Hg	128 ± 19 [461]	132 ± 23 [102]	129 ± 28 [54]	0.416	138 ± 24 [525]	134 ± 23 [74]	149 ± 25 [36]	0.002
African American	112 (23.5)	8 (7.6)	7 (12.3)	<0.001	96 (17.9)	20 (27.0)	8 (21.6)	0. 043
White, non-Hispanic	255 (53.6)	85 (81.0)	43 (75.4)		332 (62.1)	48 (64.9)	19 (51.4)	
Other	109 (22.9)	12 (9.4)	7 (12.3)		107 (20.0)	6 (8.1)	10 (27.0)	
Comorbidities and risk factors								
Obesity	324 (68.1)	80 (76.2)	47 (82.5)	0.025	424 (79.3)	52 (70.3)	28 (75.7)	0.204
Hypertension	192 (40.3)	70 (66.7)	39 (68.4)	<0.001	464 (86.7)	64 (86.5)	31 (83.8)	0.879
Hyperlipidemia	227 (47.7)	72 (68.6)	32 (56.1)	<0.001	392 (73.3)	50 (67.6)	25 (67.6)	0.474
Structural heart disease	255 (53.6)	77 (73.3)	43 (75.4)	<0.001	476 (89.0)	58 (78.4)	33 (89.2)	0.032
Atrial fibrillation	56 (11.8)	23 (21.9)	11 (19.3)	0.013	203 (37.9)	15 (20.3)	9 (24.3)	0.004
Coronary artery disease	43 (9.0)	17 (16.2)	5 (8.8)	0.084	183 (34.2)	12 (16.2)	9 (24.3)	0. 004
Chronic kidney disease	56 (11.8)	11 (10.5)	8 (14.0)	0.798	260 (48.6)	28 (37.8)	14 (37.8)	0.118
Diabetes mellitus	69 (14.5)	22 (21.0)	16 (28.1)	0.016	273 (51.0)	33 (44.6)	20 (54.1)	0.528
Pulmonary disease	75 (15.8)	21 (20.0)	13 (22.8)	0.281	217 (40.6)	25 (33.8)	13 (35.1)	0.460
Previous cardiovascular or cerebrovascular event	80 (16.8)	27 (25.7)	9 (15.8)	0.089	229 (42.8)	22 (29.7)	13 (35.1)	0.077
Cardiovascular measurements								
LV mass index,^a^ g/m^2^	82 ± 20 [471]	95 ± 22 [98]	93 ± 23 [57]	0.552	122 ± 35 [523]	115 ± 41 [71]	12 ± 38 [36]	0.266
Relative wall thickening (ratio)	0.43 ± 0.08 [471]	0.45 ± 0.07 [98]	0.47 ± 0.09 [57]	0.146	0.50 ± 0.13 [524]	0.48 ± 0.10 [71]	0.52 ± 0.10 [36]	0.196
LA volume index,^a^ mL/m^2^	28.7 ± 42.4 [164]	30.0 ± 7.8 [48]	27.7 ± 5.8 [25]	0.797	46.4 ± 15.7 [179]	39.4 ± 10.1 [27]	35.4 ± 10.7 [14]	0.414
Ejection fraction, %	63 ± 6 [476]	62 ± 6 [105]	63 ± 6 [57]	0.253	61 ± 6 [535]	63 ± 6 [74]	63 ± 6 [37]	0.636
Global longitudinal strain, %	−20 ± 2 [38]	−20 ± 0 [2]	0 [0]	0.963	−14 ± 4 [54]	−16 ± 4 [4]	−114(5 [3]	0.451
Early diastolic mitral filling velocity (E wave; ms)	74 ± 17 [473]	71 ± 17 [105]	71 ± 18 [56]	0.936	103 ± 27 [533]	98 ± 23 [73]	94 ± 27 [37]	0.528
E: A ratio	1.15 ± 0.40 [470]	0.88 ± 0.30 [104]	0.94 ± 0.39 [55]	0.397	1.58 ± 0.91 [526]	1.26 ± 0.63 [73]	1.16 ± 0.5 [36]	0.569
E-wave deceleration time (ms)	206 ± 44 [457]	235 ± 52 [103]	227 ± 63 [52]	0.335	207 ± 62 [522]	218 ± 69 [71]	201 ± 53 [36]	0.198
Septal mitral annular early diastolic tissue velocity (e’; cm/s)	8.2 ± 2.2 [466]	6.4 ± 2.0 [103]	7.2 ± 2.1 [56]	0.021	4.9 ± 1.4 [517]	6.1 ± 2.3 [72]	4.9 ± 1.5 [37]	<0.001
Septal E/e’ ratio	9.4 ± 2.5 [466]	11.9 ± 3.7 [103]	10.3 ± 2.9 [56]	0.001	22.1 ± 7.4 [517]	17.5 ± 6.1 [72]	21.1 ± 10.1 [37]	0.015
Lateral mitral annular early diastolic tissue velocity (e’; cm/s)	11.2 ± 3.8 [260]	7.5 ± 2.3 [68]	8.8 ± 2.5 [31]	0.080	6.6 ± 2.2 [339]	7.9 ± 2.8 [37]	6.48 ± 2.3 [27]	0.014
Lateral E/e’ ratio	7.2 ± 2.4 [260]	9.7 ± 2.9 [68]	8.4 ± 3.2 [31]	0.020	16.8 ± 6.2 [339]	12.8 ± 4.6 [37]	16.2 ± 8.0 [27]	0.033
Average E/e’ ratio	8.9 ± 2.5 [467]	11.1 ± 3.0 [104]	9.9 ± 2.9 [56]	0.006	20.4 ± 6.6 [523]	16.4 ± 5.7 [72]	19.4 ± 8.9 [37]	0.028
Pulmonary artery systolic pressure, mm Hg	29 ± 7 [320]	31 ± 7 [71]	32 ± 8 [40]	0.711	45 ± 14 [445]	44 ± 13 [51]	43 ± 18 [31]	0.783
Tricuspid regurgitation velocity, m/s	2.4 ± 0.3 [320]	2.5 ± 0.3 [73]	2.5 ± 0.3 [41]	0.701	3.0 ± 0.5 [448]	3.0 ± 0.5 [52]	2.9 ± 0.6 [31]	0.380
Biomarkers								
BNP, pg/mL	104 ± 140 [29]	158 ± 131 [9]	56 ± 35 [5]	0.173	724 ± 1674 [83]	437 ± 660 [13]	472 ± 330 [8]	0.959
NT-proBNP, pg/mL	366 ± 588 [53]	395 ± 375 [14]	142 ± 75 [3]	0.467	6,398 ± 10,665 [229]	5,742 ± 11,147 [24]	2,839 ± 6114 [14]	0.413
Clinical algorithms								
H2FPEF score (continuous)	37 ± 27 [476]	58 ± 25 [105]	57 ± 25 [57]	0.869	83 ± 21 [535]	65 ± 29 [74]	78 ± 21 [37]	0.002
H2FPEF: low	158 (33.2)	5 (4.8)	5 (8.8)		4 (0.8)	1 (1.4)	0 (0)	0.002
H2FPEF: high	30 (6.3)	20 (19.1)	9 (15.8)	<0.001	248 (46.4)	19 (25.7)	9 (24.3)	
H2FPEF: indeterminate	288 (60.5)	80 (76.2)	43 (75.4)		283 (52.9)	54 (73.0)	28 (75.7)	
HFA-PEFF: unlikely	287 (60.3)	26 (24.8)	20 (35.1)	<0.001	8 (1.5)	4 (5.4)	0 (0)	0.076
HFA-PEFF: probable	184 (38.7)	74 (70.5)	37 (64.9)		331 (61.9)	48 (64.9)	27 (73.0)	
HFA-PEFF: indeterminate	5 (1.1)	5 (4.8)	0 (0)		196 (36.6)	22 (29.7)	10 (27.0)	

Values are mean ± SD [N] or n (%) [N]. *P* value refers to statistical test between correct, incorrect, and unclassified groups within controls, and the same comparison within cases.

AI = artificial intelligence; BMI = body mass index; BNP = brain natriuretic peptide; H2FPEF = Heavy, Hypertensive, Atrial Fibrillation, Pulmonary Hypertension, Elder, and Filling Pressure; HFA-PEFF = Heart Failure Association-Pretest Assessment, Echocardiographic and Natriuretic Peptide Score, Functional Testing, and Final Etiology; HFpEF = heart failure with preserved ejection fraction; LA = left atrial; LAVi = left atrial volume index; LV = left ventricle; LVMi = left ventricular mass index; NT-proBNP = N-terminal pro brain natriuretic peptide; SBP = systolic blood pressure.

a Indexing was performed to body surface area. Average filling refers to the calculated mean of the septal and lateral mitral annular early diastolic tissue velocity when both metrics are available, or the available metric when only 1 is available. Categories within the “Comorbidities and risk factors” section only refer to individuals with the given condition present. Obesity refers to a BMI >25.0 kg/m^2^. Structural heart disease refers to the presence of an enlarged LA volume index (≥34.0 mL/m^2^) or LV mass index (≥116/96 g/m^2^ for males and females, respectively), a relative wall thickening >0.42, or a posterior wall thickness ≥12 mm. Pulmonary disease refers to the presence of lung disease or chronic obstructive pulmonary disorder. Previous cardio- or cerebrovascular event refers to the presence of a previous stroke, transient ischemic attack, coronary artery revascularization, or myocardial infarction. Pulmonary artery systolic pressure calculated as: 4 (tricuspid regurgitation velocity)^2^ + estimated right atrial pressure. HFA-PEFF probability categories calculated according to Pieske et al.^12^ Patients with a score of 0 or 1, between 2 and 4, and 5 or more, were denoted as unlikely, indeterminate, and probable likelihood of HFpEF, respectively. H2FPEF continuous and categorical scores were calculated according to Reddy et al.^11^ For the categorical score, patients with a score of 0 or 1, 2 to 5, or 6 to 9, were denoted as low, indeterminate, and high probability of HFpEF, respectively.

Sensitivity analyses were performed to identify whether bias in age, sex, or year of echocardiogram meaningfully influenced the classification accuracy. In all instances, sensitivity and specificity were higher than the a priori benchmarks (range: 83.7%-87.6% and range: 78.4%-82.4%, respectively) (Supplemental Appendix). Likewise, no a priori identified patient or technical factors meaningfully impacted the classification accuracy, with sensitivity and specificity maintained across subgroups (Supplemental Appendix).

#### Repeatability and reproducibility of AI HFpEF model

The model demonstrated perfect agreement for repeatability of all model outputs (Table 3). From the main testing data set, 2 separate video clips per patient were available for 34 controls and 48 cases to assess reproducibility. The model demonstrated acceptable agreement for negative diagnostic outputs (76.9%; 95% CI: 60.0%-90.3%), positive diagnostic outputs (86.7%; 95% CI: 76.9%-94.3%), and nondiagnostic outputs (45.5%; 95% CI: 13.3%-72%) (Table 3).

**Table 3:** Repeatability (Same Video Clip Used Twice), and Reproducibility (Different Video Clip Per Patient) of the AI HFpEF Model

	Read 2		
	Negative	Positive	No Classification
Repeatability			
Read 1			
Negative	550	0	0
Positive	0	640	0
No classification	0	0	94
Negative agreement (95% CI)	100		
Positive agreement (95% CI)	100		
No classification agreement (95% CI)	100		
Reproducibility			
Read 1			
Negative	20	3	3
Positive	3	39	2
No classification	3	4	5
Negative agreement (95% CI)	76.9 (60.0-90.3)		
Positive agreement (95% CI)	86.7 (76.9-94.3)		
No classification agreement (95% CI)	45.5 (13.3-72.0)		

AI = artificial intelligence; HFpEF = heart failure with preserved ejection fraction.

### Utility of AI HFpEF model and clinical scores

To assess whether the AI HFpEF model was identifying markers of diastolic dysfunction in the echocardiogram, we assessed the classification performance of guideline derived cut-points for individual echocardiographic parameters in the independent testing data set. Sensitivity (range: 52.2%-100%) and specificity (range: 47.1%-96.8%) were variable and data were often missing (range: 158-1,259 patients) (Table 4).

**Table 4:** Traditional Methods to Classify Patients as High or Low Likelihood of Having HFpEF Using Guideline Echocardiogram Cut Points or Validated Clinical Algorithms

Criteria	n	No Class	Sensitivity (95% CI)	Specificity (95% CI)
AI HFpEF model	1,284	94	87.8 (84.6–90.6)	81.9 (78.0–85.3)
Echocardiogram guideline thresholds				
Left atrial volume index^a^ ≥34 mL/m^2^	457	39	83.5 (72.5–92.0)	84.4 (73.9–94.0)
LV mass index^a^ >116/95 g/m^2^	1,256	93	65.8 (61.2–69.5)	86.6 (83.9–90.2)
Relative wall thickness >0.42	1,257	93	75.3 (71.3–78.8)	47.1 (41.4–51.5)
LV posterior wall thickness >12 mm	1,259	94	52.2 (47.3–56.5)	86.5 (83.3–89.3)
Global longitudinal strain ≥-16%	101	3	72.4 (50.0–100.0)	92.5 (16.7–100.0)
Average E/e’ ratio ≥15	1,259	93	78.2 (73.9–82.2)	96.8 (95.1–98.3)
Septal mitral annular early diastolic tissue velocity (e’) <7 cm/s	1,251	93	86.2 (82.9–90.0)	73.8 (70.1–78.2)
Lateral mitral annular early diastolic tissue velocity (e’) <10 cm/s	762	58	89.6 (84.8–94.0)	56.4 (48.6–63.3)
Septal E/e’ >15	1,251	93	81.7 (77.9–84.4)	96.8 (95.2–98.3)
Lateral E/e’>13	762	58	67.6 (60.1–74.1)	95.1 (91.5–98.2)
Tricuspid regurgitation velocity >2.8 m/s	965	72	60.2 (54.5–65.3)	90.6 (87.0–93.9)
Pulmonary artery systolic pressure >35 mm Hg	958	71	73.4 (67.5–78.4)	84.7 (81.1–89.1)
BNP/NT-proBNP ≥125/35	459	29	97.6 (94.0–100.0)	36.9 (22.6–56.6)
Clinical algorithm				
HFA-PEFF	1,284	701	95 (92.0–97.8)	97.1 (94.7–98.9)
H2FPEF	1,284	776	98.2 (96.3–99.8)	74.0 (66.9–79.0)

Data presented are the total sample of patients with data available for use in the classification (“n”), number of patients with data available who receive a nondiagnostic output from the AI HFpEF model (“no class).

H2FPEF = Heavy, Hypertensive, Atrial Fibrillation, Pulmonary Hypertension, Elder, and Filling Pressure; HFA-PEFF = Heart Failure Association-Pretest Assessment, Echocardiographic and Natriuretic Peptide Score, Functional Testing, and Final Etiology; HFpEF = heart failure with preserved ejection fraction; LA = left atrial; LV = left ventricle.

a Indexing was performed to body surface area. Average filling refers to the calculated mean of the septal and lateral mitral annular early diastolic tissue velocity when both metrics are available, or the available metric when only 1 is available. Pulmonary artery systolic pressure calculated as 4 (tricuspid regurgitation velocity)^2^ + estimated right atrial pressure (5 mm Hg). HFA-PEFF probability categories calculated according to Pieske et al^12^ Patients with a score of 0 or 1 were considered unlikely likelihood of HFpEF (negative output; predicted control), and 5 or more considered probable likelihood of HFpEF (positive output; predicted case). H2FPEF categorical scores were calculated according to Reddy et al^11^ Patients with a score of 0 or 1 were considered low probability of HFpEF (negative output; predicted control), and 6 or more considered high probability of HFpEF (positive output; predicted case).

**Figure 3:**
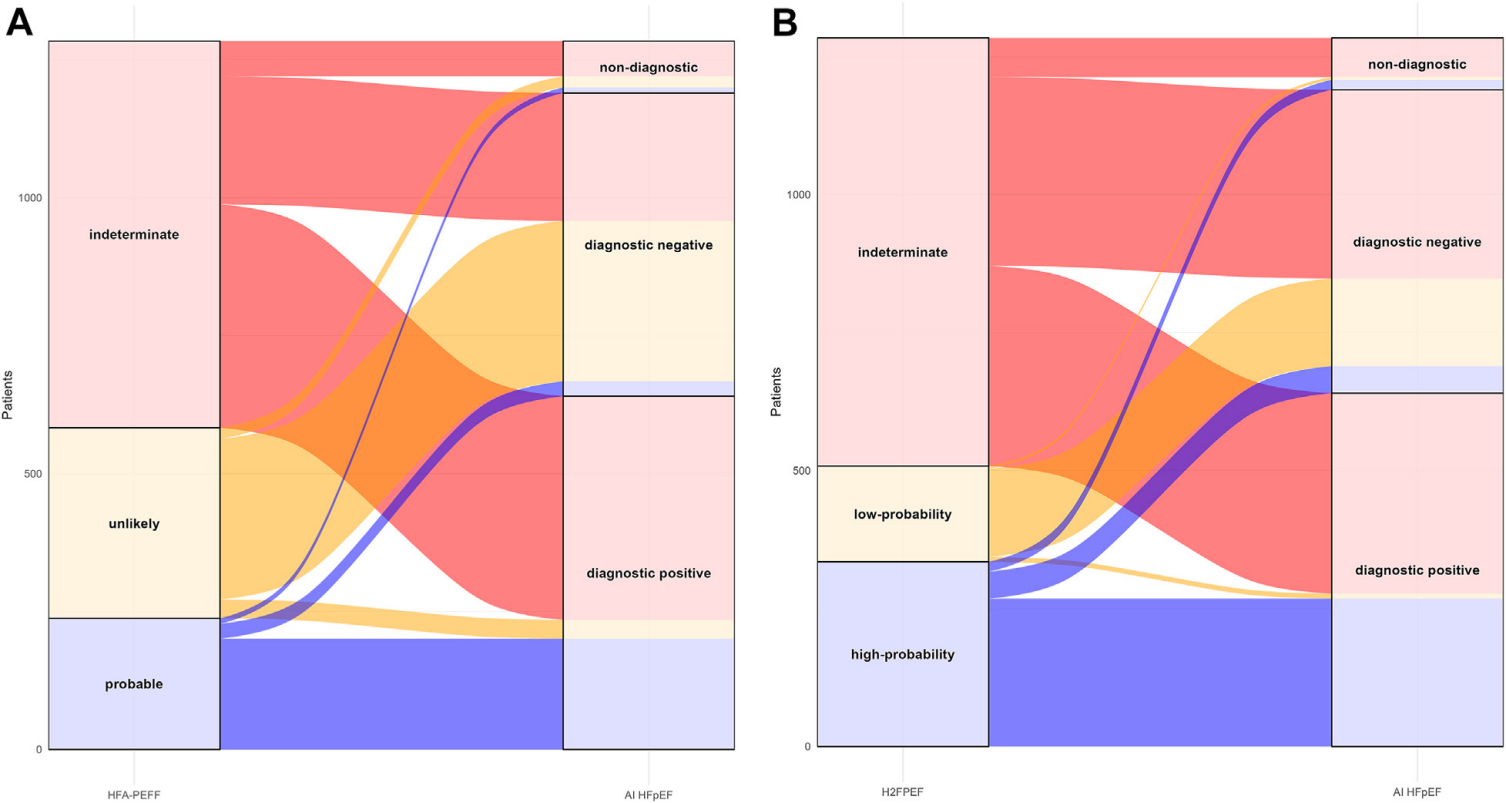
**Alluvial Plot Demonstrating Reclassification of Patients Using Clinical Scores Compared to the AI HFpEF Model** Patients in the independent testing data set were given a categorical score based on relevant functional and morphological echocardiographic, and biomarker parameters. Patients were categorized as unlikely (0 or 1), indeterminate (2-4), or probable (5-6) likelihood of heart failure with preserved ejection fraction for the Heart Failure Association-Pretest Assessment, Echocardiographic and Natriuretic Peptide Score, Functional Testing, and Final Etiology (HFA-PEFF) score **(A)**, and low-probability (0 or 1), indeterminate (2-5), or high-probability (6-9) of heart failure with preserved ejection fraction for the Heavy, Hypertensive, Atrial Fibrillation, Pulmonary Hypertension, Elder, and Filling Pressure (H2FPEF) score **(B)**. AI = artificial intelligence; HFpEF = heart failure with preserved ejection fraction.

The HFA-PEFF score had high sensitivity (95.0%; 95% CI: 92.0%-97.8%) and specificity (97.1%; 95% CI: 94.7%-98.9%) but was nondiagnostic in 701 (54.6%) patients; the AI HFpEF model successfully reclassified 515 (73.5%) of these patients (Figure 3). This resulted in a sensitivity and specificity of 87.3% (95% CI: 83.0%-90.8%) and 71.3% (95% CI: 65.0%-77.2%), respectively, of the AI HFpEF model in the reclassified patients with HFA-PEFF.

The H2FPEF score also demonstrated high sensitivity (98.2%; 95% CI: 96.3%-99.8%) and specificity (74.0%; 95% CI: 66.9%-79.0%), but similarly had high proportions of nondiagnostic outcomes (n = 776; 60.4%). Of these nondiagnostic outcomes, the AI HFpEF model successfully reclassified 571 patients (73.6%) (Figure 3), representing a sensitivity and specificity of 84.0% (95% CI: 80.2%-87.9%) and 78.3% (95% CI: 74.2%-82.9%), respectively, for the AI HFpEF model in reclassified patients with H2FPEF.

**Figure 4:**
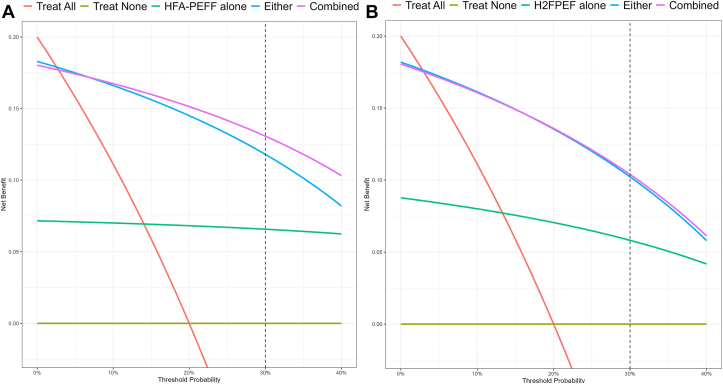
**Decision Curve Analysis for Patient Management Decisions Incorporating the AI HFpEF Model** Decision curve analysis comparing net benefit of patient management decisions are based on the output of HFA-PEFF score, **(A)** and score **(B)** and/or the artificial intelligence heart failure with preserved ejection fraction model. Assuming a 20% prevalence of heart failure with preserved ejection fraction in the target population, and a threshold probability of 30%, managing a patient based on the combined information provided by the PEFF or H2FPEF score and the artificial intelligence heart failure with preserved ejection fraction model results in 5 to 6 more patients with heart failure with preserved ejection fraction (out of assumed 20 in the population) being managed appropriately compared to managing based on the PEFF or H2FPEF scores alone. Further details are provided in the Supplemental Appendix. AI = artificial intelligence; H2FPEF = Heavy, Hypertensive, Atrial Fibrillation, Pulmonary Hypertension, Elder, and Filling Pressure; HFA-PEFF = Heart Failure Association-Pretest Assessment, Echocardiographic and Natriuretic Peptide Score, Functional Testing, and Final Etiology; HFpEF = heart failure with preserved ejection fraction.

The implications of patient management decisions were compared between current clinical practice (HFA-PEFF and H2FPEF scores), or utilizing information gleaned from the AI HFpEF model. Consistent with the proposed use case of the model, using the combined information from the AI HFpEF model and existing clinical scores in patient management decisions resulted in up to 6/20 more patients being managed correctly than based on existing scores alone (Figure 4, Supplemental Appendix).

### AI HFpEF model and clinical endpoints

In the testing data set, 22 patients were referred for right heart catheterization within 1 year of the TTE (median: 45 [IQR: 7-161] days; 19 cases, 3 controls). For cases, 16 were confirmed HFpEF (rest pulmonary capillary wedge pressure ≥15 mm Hg), all of whom were classified as HFpEF by AI (100% sensitivity). In the remaining 3 patients, diagnoses included cardiac amyloidosis, coronary artery disease, and pulmonary hypertension. For the 3 controls, none had HFpEF by catheterization (two-thirds correct by AI; 67% specificity). In the testing data set, estimated 5-year mortality was 37.2% (95% CI: 33.8%-40.5%; 444 deaths), with significant differences in risk of mortality between classification groups according to the AI output after adjustment for age (Central Illustration).

## Discussion

We have developed and validated a novel AI model that, using only a single A4C video clip, demonstrated excellent ability to distinguish between patients with and without HFpEF. Compared to current clinical algorithms, the AI HFpEF model resulted in an accurate diagnostic output for more patients, and successfully identified patients with worse 5-year survival. The ability to automatically detect HFpEF with limited clinical information has important practical ramifications, particularly for screening in centers without the time or expertise to complete diagnostic quality diastolic assessment, resulting in indeterminate or unclear clinical diagnoses. Combined, the technical and clinical feasibility demonstrated with this model could result in faster patient access to effective pharmacological therapy.

Although there have been other AI models developed to tackle the burden of HFpEF, they have relied on accurate chamber segmentation to derive a series of features used for classification^19^ or used the complete echocardiographic study to automate computation of left ventricular diastolic function parameters.^18^ To our knowledge, this is the first model developed on a single routinely acquired video clip, demonstrating feasibility and high classification accuracy consistent with comprehensive clinical and echocardiographic assessment in expert centers,^7^^,^^23^^,^^24^ albeit using substantially less clinical information. In comparison, recent AI developments in diastolic function scoring^17^ were developed using complete data sets; a scenario rarely representing the clinical norm. Comparative effectiveness of different models is beyond the scope of this study, but considering the observed proportion of missing data (Table 4), such a model could support existing diagnostic efforts without requiring additional calculation of current or new (eg, left atrial strain) metrics.

The phenomenon of nondiagnostic outcomes using existing guidelines and the sometimes cumbersome intricacy of diastolic assessment in HFpEF are widely reported.^7^^,^^14^^,^^24^, ^25^, ^26^ The performance of such methods varies considerably,^7^^,^^11^^,^^23^^,^^24^ but can be excellent in expert centers, or when missing or discordant data are not an issue. In complex clinical cases, whilst there is guidance for estimating filling pressure when echocardiographic signals are difficult to interpret (atrial fibrillation^10^), the assessment is often avoided entirely. Compared to current clinical algorithms, or guideline-derived cut-offs for various diagnostic markers, the AI HFpEF model retuned fewer nondiagnostic outputs (Table 4), successfully reclassifying almost 75% of those who would be non-diagnostic according to the HFA-PEFF or H2FPEF scores (Figure 3). Furthermore, the model identified those with increased risk of mortality (Central Illustration), and its use in clinical practice—particularly in those who would otherwise be indeterminate—might facilitate a higher proportion of patients being managed correctly (Figure 4, Supplemental Appendix). Further research is required to understand whether the added feasibility and high classification performance translate to meaningful clinical endpoints, including reductions in follow-up procedures, hospitalization, or death.

Technological advances provide increased capacity to capture information not readily observed by the human eye, albeit often at the expense of interpretability. Grad-CAM is one approach to facilitate interpretability in AI, identifying important regions in the image to discriminate between cases and controls. In an example of correct classification (Figure 2), the Grad-CAM highlights regions which correspond to clearly defined cardiac structures which might have clinical importance.^9^^,^^10^^,^^12^ In incorrect classifications, seemingly extracardiac structures would serve as a red flag for clinicians in their trust of the model output. Specifically, the Grad-CAM should prompt the clinician to consider whether the highlighted region is important in the clinical discrimination between HFpEF and not HFpEF; if so, the model output could be trusted and follow-up management initiated (testing and/or prescription), if not, the output requires further validation. This fits with the intention for such a preliminary (categorical) model in clinical application, acting as another reader, encouraging clinicians to take a second look if required, or perform follow-up testing if necessary. Nonetheless, while a high-level of “explainability” might or might not facilitate greater benefit to patients, future work is required to better understand such models and guide more transparent and patient-level interpretation.

Comparison of correct, incorrect, and nonclassified patients highlight that the model has excellent discriminatory capacity (Central Illustration), particularly in “typical” HFpEF compared to more complex differential diagnoses (Table 2). Misclassified patients might represent a cohort demonstrating provokable increases in filling pressure, or signs and symptoms of HF not captured by the clinical coding employed herein. Important validation work in the future will involve assessment of model performance in adjudicated HF outcomes and/or invasively measured filling pressure.

### Study limitations

The diagnostic details of each case were not adjudicated. Therefore, it is possible that some controls had subclinical disease, albeit representative of patients in major clinical trials (Supplemental Appendix). Nonetheless, an important progression for the current model is to increase capacity and validate detection of HFpEF earlier in the clinical pathway, particularly when patients might have dyspnea on exertion, but not at rest (eg, patients referred for diastolic stress testing, or invasive filling pressure measurements at rest and with exertion^9^^,^^12^), or when limited echocardiographic imaging occurs earlier in the pathway (eg, point-of-care ultrasound). Another limitation is that complete matching for age was not possible; patients with HFpEF were older. However, survival analysis was age-adjusted and sensitivity analysis demonstrated no meaningful change in interpretation in only age-matched patients. Future work will be required for recalibration or updating of the model in other patient groups (eg, increased filling pressure but no HF diagnosis, or indeterminate filling pressure assessment by TTE), validating its application in other echocardiography laboratories and in different demographic groups, and prospective evaluation of comparative effectiveness with clinical scores.

## Conclusions

We present a novel AI HFpEF model which, based on only a single routinely acquired TTE video clip, accurately detected HFpEF, provided fewer nondiagnostic outputs than current clinical scores, and identified patients with worse survival. The application of this classifier in the screening for HFpEF, particularly when their diagnosis is uncertain, has the potential to automate an accurate detection process for a complex clinical syndrome, resulting in more patients getting a correct and expeditious diagnosis.

PERSPECTIVES **COMPETENCY IN MEDICAL KNOWLEDGE:** A 3D CNN was developed to identify patients with HFpEF using only the A4C echocardiogram video clip. Age-adjusted mortality was higher in patients identified as having HFpEF.

**TRANSLATIONAL OUTLOOK:** Future work is needed to assess the model in other patient groups (eg, increased filling pressure but no HF diagnosis or indeterminate filling pressure assessment by echocardiography), and validate its application in other echocardiography laboratories and in different demographic groups.

## Funding support and author disclosures

This study was supported by grants from the 10.13039/100005400American Society of Echocardiography (ASE) Foundation and Ultromics Ltd. Dr Pellikka is supported as the Betty Knight Scripps Professor of Cardiovascular Disease Clinical Research. Drs Akerman, Porumb, Beqiri, Chartsias, Hawkes, Gomez, Author Sarwar, O'Driscoll, Author Leeson, Upton, and Woodward are employed by Ultromics Ltd. All other authors have reported that they have no relationships relevant to the contents of this paper to disclose.
