# Supplementary material for: Automated Echocardiographic Detection of Heart Failure With Preserved Ejection Fraction Using Artificial Intelligence
Source: JACC Adv. 2023 Jul 28;2(6):100452. doi: 10.1016/j.jacadv.2023.100452 (PMC11198161; doi:10.1016/j.jacadv.2023.100452)
Supplement: Supplementary data [file mmc1.docx]

**SUPPLEMENTAL APPENDIX**

# Methods: Data sources and Study Population

*Independent testing of the AI HFpEF model*

Independent retrospective data were collected within Mayo Clinic Health System to test the AI HFpEF model. Patients were selected from geographically distinct areas from the data used in model training and validation to ensure generalizability and representativeness with the intended use population. Data were selected from clinical sites spanning four states, and outreach services across five states.

Data selection at the individual sites, to reach the required sample size (detailed below), was achieved with an approximately proportional sampling relative to the geographical area. The planned and actual proportional sampling from these independent sites is outlined in Supplemental Table 1 below.

**Supplemental Table 1**. Data sampling for independent testing dataset

| Sites | Population  (2020 Census) | Target Proportion | Actual Proportion |
| --- | --- | --- | --- |
| **Large Clinical Site** |  |  |  |
| Red Wing (MN) | 16,459 | 1-3% | 1.0% |
| Albert Lea (MN) | 18,492 | 1-3% | 3.5% |
| Austin (MN) | 26,174 | 3-6% | 6.8% |
| Mankato (MN) | 44,488 | 5-10% | 13.5% |
| La Crosse (WI) | 139,627 | 10-15% | 8.7% |
| Scottsdale (AZ) | 241,361 | 15-25% | 19.2% |
| Jacksonville (FL) | 902,488 | 40-70% | 34.0% |
| **Total Large Clinical Sites** | 1,389,089 |  |  |
| **Community Outreach Services**  **(AZ, FL, IA, MN, WI)** |  | 5-10%  Approximately equal to median size site (Mankato) | 13.5% |

Patient medical record databases and echocardiographic databases were screened for individuals meeting the ground truth criteria for HFpEF (cases), with proportional sampling at each site relative to the approximate geographical area (for the large clinical sites). The proportion of patients randomly sampled from the large outreach centre was intended to represent the median site for the larger clinical centres. **Note**: AZ, Arizona; FL, Florida; IA, Iowa; MN, Minnesota; WI, Wisconsin.

# Methods: Identification of sub-groups

*Clinical diagnosis of heart failure*

Documented clinical diagnosis of HF, based on an International Classification of Diseases (ICD) 9 or 10 code, within one year of the associated echocardiogram (case) or lack of this diagnosis (control) was collected from the patient medical records. A HF diagnosis was determined by the attending clinician in the patients’ medical records. Representative ICD codes are provided in Supplemental Table 2 below.

**Supplemental Table 2**. International Classification of Diseases (ICD) codes used to indicate heart failure (HF) in ground truth determination of heart failure with preserved ejection fraction (HFpEF)

| ICD-9 Code | Description | ICD-10 Code | Description |
| --- | --- | --- | --- |
| I50.1 | Left ventricular failure | 428 | Heart Failure |
| I50.20 | Unspecified systolic (congestive) heart failure | 428.0 | Congestive Heart Failure, Unspecified |
| I50.21 | Acute systolic (congestive) heart failure | 428.1 | Left Heart Failure |
| I50.22 | Chronic systolic (congestive) heart failure | 428.2 | Systolic heart failure |
| I50.23 | Acute on chronic systolic (congestive) heart failure | 428.20 | Unspecified systolic heart failure |
| I50.30 | Unspecified diastolic (congestive) heart failure | 428.21 | Acute systolic heart failure |
| I50.31 | Acute diastolic (congestive) heart failure | 428.22 | Chronic systolic heart failure |
| I50.32 | Chronic diastolic (congestive) heart failure | 428.23 | Acute on chronic systolic heart failure |
| I50.33 | Acute on chronic diastolic (congestive) heart failure | 428.3 | Diastolic heart failure |
| I50.40 | Unspecified combined systolic (congestive) and diastolic (congestive) heart failure | 428.30 | Unspecified diastolic heart failure |
| I50.41 | Acute combined systolic (congestive) and diastolic (congestive) heart failure | 428.31 | Acute diastolic heart failure |
| I50.42 | Chronic combined systolic (congestive) and diastolic (congestive) heart failure | 428.32 | Chronic diastolic heart failure |
| I50.43 | Acute on chronic combined systolic (congestive) and diastolic (congestive) heart failure | 428.33 | Acute on chronic diastolic heart failure |
| I50.8 | Other heart failure | 428.4 | Syst/Diastolic Hrt Fail |
| I50.81 | Right heart failure | 428.40 | Unspec combined systolic & diastolic heart failure |
| I50.810 | Right heart failure, unspecified | 428.41 | Acute comb systolic & diastolic heart failure |
| I50.811 | Acute right heart failure | 428.42 | Chronic comb systolic & diastolic heart failure |
| I50.812 | Chronic right heart failure | 428.43 | Acute on chronic systolic & diastolic heart failure |
| I50.813 | Acute on chronic right heart failure | 428.9 | Heart failure unspecified |
| I50.814 | Right heart failure due to left heart failure |  |  |
| I50.82 | Biventricular heart failure |  |  |
| I50.83 | High output heart failure |  |  |
| I50.84 | End stage heart failure |  |  |
| I50.89 | Other heart failure |  |  |
| I50.9 | Heart failure, unspecified |  |  |

*Transthoracic echocardiograms*

Transthoracic echocardiogram (TTE) data were collected by an appropriately credentialed cardiac sonographer and comprised multiple methods of quantification. All studies were interpreted by cardiologists with advanced training in echocardiography according to usual clinical practice.

Manufacturer instructions and local standard clinical protocols were used for image acquisition. All echocardiograms were performed on a variety of available ultrasound systems (GE Healthcare, Philips Medical Systems, and Siemens) to facilitate generalizability. Apical four chamber views were de-identified and exported as Digital Imaging and Communications in Medicine (DICOM) files.

*Preserved systolic function*

Left ventricular (LV) ejection fraction (EF) was determined by quantitative two-dimensional biplane volumetric Simpson’s method from four and two chamber views or if this was unavailable, by monoplane four-chamber method, or by two-dimensional echocardiography using the modified Quinones formula from the parasternal views, or when image quality was insufficient for measurement, by visual estimate per guidelines ^1,2^.

*Evidence of elevated intra-cardiac filling pressure*

Data from a comprehensive TTE satisfying the evidential burden for increased intra-cardiac filling pressure represented diastolic dysfunction indicated by an elevated average early diastolic mitral filling velocity (E) to early diastolic mitral tissue velocity (e’) ratio (E/e’), decreased septal e’, decreased lateral e', increased tricuspid regurgitation velocity, elevated estimated pulmonary artery systolic. Collection and interpretation of echocardiographic indices providing evidence of increased intracardiac filling pressure were according to relevant guidelines ^3,4^. According to guidelines^4^, intracardiac filling pressure considered increased for individuals with grade 2 or greater degree of diastolic dysfunction.

# Methods: Overview of the AI HFpEF model

*AI HFpEF model outputs*

A non-diagnostic output was generated based on model uncertainty, using the expected entropy on all predictions across the consecutive sequences of 30 frames. The threshold for expected entropy (0.59) was determined according to the threshold at which classification performance was improved significantly without omitting more than 10% of the data during model training (Supplemental Figure 1).


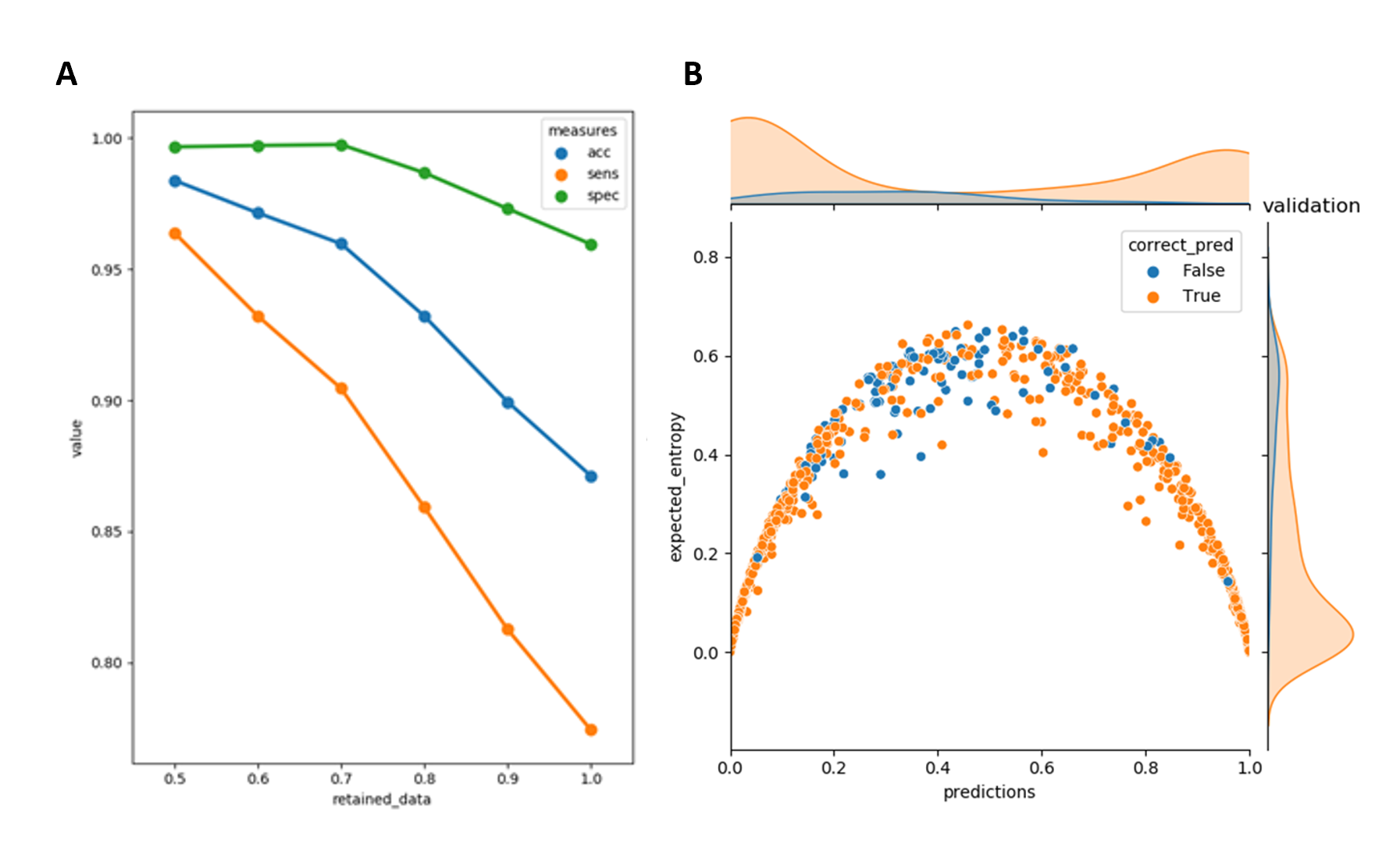


**Supplemental Figure 1**. Data from model development (validation) used to determine the threshold applied to subsequent un-seen data for high uncertainty (expected entropy) and thus a non-diagnostic output. Panel A refers to a retention plot in which the most uncertain data (highest expected entropy) are removed in 10% iterations (x-axis), with performance of the model after the data are removed (accuracy, acc; sensitivity, sens; specificity, spec; y axis). When 10% of the most uncertain data are removed, this corresponds to an expected entropy threshold of 0.59, and significantly increases performance and keeping data removal at a clinically justifiable level. Panel B refers to a plot of prediction class probability (x-axis) and expected entropy (y-axis) with correct (orange) and incorrect (blue) classifications according to the AI HFpEF model highlighted. Combined, these plots highlight that application of a 0.59 expected entropy threshold resulted in an acceptable level of data removed, and improved performance of the model after removal of a high proportion of misclassified patients.

# Methods: Statistical Analysis

Once a final model had been developed as above, measures of calibration and classification performance were assessed. Calibration of the predicted probabilities with empirical probabilities (observed proportions obtained from the ground truth label) was assessed using the Hosmer-Lemeshow goodness-of-fit test ^5^. Gradient-weighted class activation mapping (Grad-CAM) method was employed for visualising the regions in the input images that were used by the model for outputting the final prediction class. Classification performance was assessed according to area under ROC (AUROC), sensitivity, and specificity ^6,7^. The impact of incorporating the AI HFpEF model into current clinical practice was assessed using decision curve analysis ^8^.

*AI HFpEF model independent testing: Sample size determination and analysis*

To test the hypothesis that the classification accuracy of the AI HFpEF model was superior to current clinical practice, we compared the classification sensitivity and specificity to those of previously reported data. Classification according to the ground truth determination (case and control) were compared to the output of the AI HFpEF model in a 2x2 confusion matrix, omitting the data with a non-diagnostic output, and subsequently calculating sensitivity, specificity, and predictive values. Statistical comparison to literature reported performance was conducted using a one-sided Binomial exact test (alpha = 0.025).

We conducted a systematic search of the literature to identify diagnostic accuracy of imaging and biomarkers assessment in HFpEF. From this search, seven key publications^9–15^ were identified, using 37 imaging and clinical biomarkers assessment, representing approximately 3,100 patients enrolled at sites predominantly in the USA, and referred for TTE and/or right heart catheterisation due to unexplained dyspnea or suspected HF.

The average reported sensitivity and specificity were 74% and 65%, respectively, albeit with large heterogeneity. Given that these data likely represent a selection bias in the types of medical institutions diagnosing HFpEF (i.e., expert sites will be more accurate, but less expert sites less so, as per the central objective of the study), we believed that the average data would represent a valid compromise for estimating current clinical practice. This approach has also been chosen in acknowledgment of recent meta-analyses on the topic ^16^, that cites a high risk of bias in HFpEF meta-analyses due to patient selection and heterogeneity across the literature (I2 >90%).

A summary of the relevant studies contributing the null hypothesis rationale are highlighted below:

**Reddy et al., (2018), A simple evidence-based approach to help guide diagnosis of HFpEF** ^9^

- Patient population: patients with concurrent TTE and invasive right or left heart catheterisation (RHC, or LHC, respectively) between 2006 and 2016.
- Comparison of interest: Clinical diagnosis of HFpEF using all available information and multiple clinical readers, compared to single echocardiographic measurements, 2016 algorithm, and newly developed H2FPEF score.
- **Reported Diagnostic Performance:**
  - Sensitivity: 57%, 46%, 76%, and 78% for 2016 guidelines derivation cohort, 2016 guidelines validation cohort, H2FPEF derivation cohort, and H2FPEF validation cohort, respectively.
  - Specificity: 78%, 90%, 78%, and 84% for 2016 guidelines derivation cohort, 2016 guidelines validation cohort, H2FPEF derivation cohort, and H2FPEF validation cohort, respectively.

**Aizpurua et al. (2020), Validation of the HFA-PEFF score for the diagnosis of HFpEF** ^10^

- Patient population: Patients referred to medical centre for suspected HFpEF.
- Comparison of interest: Consensus and holistic clinical determination of HFpEF vs. HFA-PEFF score and related components.
- **Reported Diagnostic Performance:**
  - Sensitivity: 69%, 99%, 88%, 75%, 95%, 76%, 93%, and 73% for HFA-PEFF rule in, HFA-PEFF rule-out, Functional score rule-in, Functional score rule-out, Morphological score rule-in, Morphological score rule-out, Biomarker score rule-in, and Biomarker score rule-out, respectively.
  - Specificity: 93%, 19%, 29%, 33%, 29%, 79%, 83%, and 88% for HFA-PEFF rule in, HFA-PEFF rule-out, Functional score rule-in, Functional score rule-out, Morphological score rule-in, Morphological score rule-out, Biomarker score rule-in, and Biomarker score rule-out, respectively.
  - Negative predictive value: 64%, 73%, 30%, 80%, 50%, 62%, 67%, and 63% for HFA-PEFF rule in, HFA-PEFF rule-out, Functional score rule-in, Functional score rule-out, Morphological score rule-in, Morphological score rule-out, Biomarker score rule-in, and Biomarker score rule-out, respectively.
  - Positive predictive value: 98%, 87%, 87%, 86%, 88%, 95%, 97%, and 97% for HFA-PEFF rule in, HFA-PEFF rule-out, Functional score rule-in, Functional score rule-out, Morphological score rule-in, Morphological score rule-out, Biomarker score rule-in, and Biomarker score rule-out, respectively.

**Wijk et al. (2020), The HFA-PEFF and H2FPEF scores largely disagree in classifying patients with suspected HFpEF** ^11^

- Patient population: Patients with suspected HFpEF
- Comparison of interest: Clinical diagnosis of HFpEF vs. H2FPEF or HFA-PEFF
- **Reported Diagnostic Performance:**
  - Sensitivity: 52.7% and 70% (H2FPEF and HFA-PEFF, respectively)
  - Specificity: 82.5% and 90.5% (H2FPEF and HFA-PEFF, respectively)
  - Negative predictive value: 26.8% and 38.8% (H2FPEF and HFA-PEFF, respectively)
  - Positive predictive value: 93.5% and 97.2% (H2FPEF and HFA-PEFF, respectively)

**Parcha et al. (2021), Diagnostic and prognostic implications of HFpEF scoring systems** ^12^

- Patient population: Re-analysis of multiple trials
- Comparison of interest: TOPCAT, RELAX, and Ambulatory Cohort using clinical diagnosis of HFpEF vs. HFA-PEFF and H2FPEF
- **Reported Diagnostic Performance:**
  - Sensitivity: 83.6%, 100%, 55.9%, 99.5%, 26.4%, 98.8%, 64%, 100%, 69.1%, 99.6%, 57.4%, and 99.4% for TOPCAT HFA-PEFF algorithm rule-in, TOPCAT HFA-PEFF algorithm rule-out, TOPCAT H2FPEF algorithm rule-in, TOPCAT H2FPEF algorithm rule-out, RELAX HFA-PEFF algorithm rule-in, RELAX HFA-PEFF algorithm rule-out, RELAX H2FPEF algorithm rule-in, RELAX H2FPEF algorithm rule-out, Ambulatory HFA-PEFF algorithm rule-in, Ambulatory HFA-PEFF algorithm rule-out, Ambulatory H2FPEF algorithm rule-in, and Ambulatory H2FPEF algorithm rule-out, respectively.
  - Specificity: 78.2%, 9.7 %, 92.5%, 8%, 78.2%, 9.7%, 92.5%, 8%, 76.8%, 8.6%, 89.7%, and 7.7% for TOPCAT HFA-PEFF algorithm rule-in, TOPCAT HFA-PEFF algorithm rule-out, TOPCAT H2FPEF algorithm rule-in, TOPCAT H2FPEF algorithm rule-out, RELAX HFA-PEFF algorithm rule-in, RELAX HFA-PEFF algorithm rule-out, RELAX H2FPEF algorithm rule-in, RELAX H2FPEF algorithm rule-out, Ambulatory HFA-PEFF algorithm rule-in, Ambulatory HFA-PEFF algorithm rule-out, Ambulatory H2FPEF algorithm rule-in, and Ambulatory H2FPEF algorithm rule-out, respectively.
  - Negative predictive value: 86.3%, 100%, 79%, 96.7%, 81.6%, 97.2%, 92.5%, 100%, 71.3%, 95.2%, 73.6%, and 94.7% for TOPCAT HFA-PEFF algorithm rule-in, TOPCAT HFA-PEFF algorithm rule-out, TOPCAT H2FPEF algorithm rule-in, TOPCAT H2FPEF algorithm rule-out, RELAX HFA-PEFF algorithm rule-in, RELAX HFA-PEFF algorithm rule-out, RELAX H2FPEF algorithm rule-in, RELAX H2FPEF algorithm rule-out, Ambulatory HFA-PEFF algorithm rule-in, Ambulatory HFA-PEFF algorithm rule-out, Ambulatory H2FPEF algorithm rule-in, and Ambulatory H2FPEF algorithm rule-out, respectively.
  - Positive predictive value: 74.4%, 45.7%, 80.7%, 37.6%, 22.5%, 18.4%, 64%, 18.4%, 74.9%, 52.1%, 80.8%, and 44.9% for TOPCAT HFA-PEFF algorithm rule-in, TOPCAT HFA-PEFF algorithm rule-out, TOPCAT H2FPEF algorithm rule-in, TOPCAT H2FPEF algorithm rule-out, RELAX HFA-PEFF algorithm rule-in, RELAX HFA-PEFF algorithm rule-out, RELAX H2FPEF algorithm rule-in, RELAX H2FPEF algorithm rule-out, Ambulatory HFA-PEFF algorithm rule-in, Ambulatory HFA-PEFF algorithm rule-out, Ambulatory H2FPEF algorithm rule-in, and Ambulatory H2FPEF algorithm rule-out, respectively.

**Ouwerkerk et al. (2020), Heart failure with preserved ejection fraction diagnostic scores in an Asian population** ^13^

- Patient population: Re-analysis of two Asian trials
- Comparison of interest: SHOP, and ATTRACT samples using clinical diagnosis of HFpEF vs. HFA-PEFF and H2FPEFF
- **Reported Diagnostic Performance:**
  - Sensitivity: 73.8%, 24.9%, 57.4%, and 47.5% for SHOP HFA-PEFF, SHOP H2FPEF, ATTRACT HFA-PEFF, and ATTRACT H2FPEF, respectively.
  - Specificity: 81.3%, 99.6%, 91.2%, and 98.2% for SHOP HFA-PEFF, SHOP H2FPEF, ATTRACT HFA-PEFF, and ATTRACT H2FPEF, respectively.
  - Negative predictive value: 78.4%, 60.9%, 50.0%, and 46.7% for SHOP HFA-PEFF, SHOP H2FPEF, ATTRACT HFA-PEFF, and ATTRACT H2FPEF, respectively.
  - Positive predictive value: 77.1%, 98.3%, 93.3%, and 98.3% for SHOP HFA-PEFF, SHOP H2FPEF, ATTRACT HFA-PEFF, and ATTRACT H2FPEF, respectively.

**Obokata et al. (2017), Role of diastolic stress testing in evaluation of HFpEF: simultaneous invasive-echo study** ^14^

- Patient population: Patients referred for invasive RHC or LHC due to exertional dyspnoea of unclear cause.
- Comparison of interest: Clinical diagnosis of HFpEF vs. ASE/EACVI 2016 guidelines algorithm A.
- **Reported Diagnostic Performance:**
  - Sensitivity: 34%
  - Specificity: 83%
  - Negative predictive value: 53%
  - Positive predictive value: 94%

**Anderson et al. (2019), Estimating left ventricular filling pressure by echocardiography** ^15^

- Patient population: Patients referred for RHC or LHC from international multi-site clinics due to exertional dyspnoea and multiple cardiac causes.
- Comparison of interest: Clinical diagnosis of HFpEF vs. ASE/EACVI 2016 guidelines
- **Reported Diagnostic Performance:**
  - Sensitivity: 87%
  - Specificity: 88%
  - Negative predictive value: 83%
  - Positive predictive value: 91%

To test the hypothesis that the developed AI HFpEF model would exceed these performance benchmarks by at least 5% (i.e., alternative hypothesis for sensitivity and specificity of 79% and 70%, respectively), with a 1:1 case:control rate, we determined that 324 successful trials out of 409 total trials would be required to reject the null hypothesis for sensitivity, and 287 successful trials out of 409 total trials would be required to reject the null hypothesis for specificity. Assuming each test as an independent hypothesis, the total sample size was therefore determined to be 820 patients. Given the engineered non-diagnostic output from the AI HFpEF model based on model uncertainty (see above), and a systematic search of the literature to determine average non-diagnostic outcomes (see below), we conservatively accounted for an extra 21.9% of patients each for sensitivity and specificity, thus requiring a total of 1048 patients.

A summary of the relevant studies contributing the anticipated non-diagnostic outputs are highlighted below:

**Lancelloti et al (2017), Echo-doppler estimation of LV filling pressure: results of multicentre EACVI euro-filling study** ^17^

- Patient population: Patients referred for invasive right heart catheterization and clinically indicated coronary angiography due to diagnosed or suspected coronary artery disease.
- **Proportion of indeterminates:**
  - Indeterminate via 2016 guidelines (HFpEF): 17%

**Wijk et al. (2020), The HFA-PEFF and H2FPEF scores largely disagree in classifying patients with suspected HFpEF study** ^11^

- Patient population: Patients with suspected HFpEF tested for performance between H2FPEF and HFA-PEFF algorithms
- **Proportion of indeterminates:**
  - Indeterminate via HFA-PEFF algorithm: 47.9%
  - Indeterminate via H2FPEF algorithm: 36.6%

**Obokata et al. (2017), Role of diastolic stress testing in evaluation of HFpEF: simultaneous invasive-echo study** ^14^

- Patient population: Patients referred for RHC due to unexplained dyspnea and suspected HFpEF.
- **Proportion of indeterminates:**
  - Indeterminate via 2016 guidelines (HFpEF): 24%
  - Indeterminate via 2016 guidelines (Non cardiac dyspnea): 10%

**Pak et al. (2022), Diagnostic accuracy of an integrated echocardiographic algorithm to estimate left ventricular filling pressure** ^18^

- Patient population: HFpEF patients undergoing first time catheter ablation for atrial fibrillation
- **Proportion of indeterminates:**
  - Indeterminate via 2016 guidelines algorithm A (not enough parameters): 17.54%
  - Indeterminate via 2016 guidelines algorithm A (indeterminate): 8.22%
  - Indeterminate via 2016 guidelines algorithm B (undetermined LAP): 7.75%

**Van de Bovenkamp et al. (2021), Validation of the 2016 ASE/EACVI guidelines for diastolic dysfunction in patients with unexplained dyspnoea** ^19^

- Patient population: Patients evaluated for unexplained dyspnoea or pulmonary hypertension with echocardiography and RHC.
- **Proportion of indeterminates:**
  - Indeterminate via 2016 guidelines algorithm A (indeterminate): 1.96%
  - Indeterminate via 2016 guidelines algorithm B (indeterminate): 18.14%
  - Indeterminate via ASE/EACVI guidelines (not applicable or indeterminate): 30%

**Nikorowitsch et al. (2021), Applying the ESC 2016, H2FPEF, and HFA-PEFF diagnostic algorithms for HFpEF in the general population** ^20^

- Patient population: ~10,000 first comers in population-based study in Hamburg resulting in final sample size of 407
- **Proportion of indeterminates:**
  - Indeterminate via ESC 2016 guidelines: 2.9%
  - Indeterminate via H2FPEF algorithm: 7.4%
  - Indeterminate via HFA-PEFF algorithm: 12.0%

**van de Bovenkamp et al. (2022), The value of passive leg raise during right heart catheterisation in diagnosing HFpEF** ^21^

- Patient population: All comers completing echocardiography and RHC for unexplained dyspnoea.
- **Proportion of indeterminates:**
  - Indeterminate via H2FPEF algorithm (Non-HFpEF): 46%
  - Indeterminate via H2FPEF algorithm (Manifest HFpEF): 54%

**Verbrugge et al. (2021), Diagnostic scores predict morbidity and mortality in patients hospitalised for HFpEF** ^22^

- Patient population: Patients with HFpEF admitted for unequivocal decompensated HF, with echo data available within 1 year of hospitalisation
- **Proportion of indeterminates:**
  - Indeterminate via HFA-PEFF algorithm: 21.0%

**Nielsen et al. (2019), Echocardiographic sub-types of heart failure in consecutive hospitalised patients with dyspnoea** ^23^

- Patient population: Patients admitted with acute dyspnoea to acute medical unit.
- **Proportion of indeterminates:**
  - Indeterminate via ESC guidelines (HFpEF): 31.4%

***AI HFpEF model independent testing: sub-group analyses***

A priori determined sub-groups of interest related to clinically meaningful patient demographics, clinical, and echocardiographic criteria. These included factors which were believed to impact the echocardiogram per se (technical), or discriminating patient level factors for cases versus controls. Specifically, they were:

- **Impact of disease prevalence** (1-52%)
- **Impact of imbalance on age, sex, and year of echocardiogram** (see below)
- **Year of echocardiogram** (2009-2021)
- **Manufacturer of echocardiogram machine** (GE Healthcare, Philips Medical Systems, Siemens)
- **Frames per cardiac cycle** (<30, ≥30)
- **Quality of 4 chamber view** (Any irregular rhythm, overall 4 chamber quality, appropriateness of gain, and endocardial border definition)
- **Geographical location** (Albert Lea, Austin, Jacksonville, La Crosse, Mankato, Red Wing, Scottsdale, Outreach Centre)
- **Sex** (male, female)
- **Age class** (<25 years, 25-54 years, 55-64 years, ≥65 years)
- **Race** (Black, White, Other)
- **Obesity** (>25 kg/m2)
- **Presence or absence of relevant comorbidities:** Hyperlipidemia, Hypertension, Structural heart disease, Atrial fibrillation, Coronary Artery Disease, Chronic Kidney Disease, Diabetes Mellitus, Pulmonary Disease, Previous Cardio- or Cerebro-vascular Event

To test the impact of disease prevalence on the AI HFpEF model performance, we completed random resampling of the full dataset, and bootstrapping with 100 iterations for each level of prevalence. Prevalence ranged from 1% to the prevalence of the full dataset (~52%), in 1% increments, to represent extremely conservative to fully enriched disease prevalence. At each level of prevalence, a random number of controls were selected with a size equal to 80-100% of the total dataset size. A random sample of cases were then selected to equal the given prevalence of disease, and classification performance was assessed in this cohort. This process was completed 100 times for each disease prevalence level, with mean and 95% confidence intervals calculated from the 100 iterations.

To test the impact of any imbalance introduced by incomplete case:control matching, we completed random resampling of the full dataset to ensure that cases and controls were exactly matched for all permutations of the three key matching criteria (age, sex, year of echocardiogram), resulting in seven matched datasets. In each dataset (associated with a given permutation of matching factors), each case was exactly matched with a control. Consistent with the sampling method described above, classification performance was assessed across iterations of random sampling.

For all other sub-group analyses, patients were split into groups according to descriptive statistics, or presence/absence of clinically relevant comorbidities. Classification performance was then assessed as per the description below.

# Methods: Clinical Risk Scores

HFA-PEFF Score

The categorical score for HFA-PEFF was calculated according to Pieske et al.^24^. Specifically, for categories of functional, structural, and biomarker evidence, major (2 point) and minor (1 point) criteria were assessed for each patient, with a maximum of 6 points in total (2 per category). These criteria are detailed below. The application of the HFA-PEFF score was limited to the second step of the published algorithm. The first step represents the development of a pre-test likelihood of HFpEF based on presenting information and immediate clinical steps. The second step represents the development of a score for categorising likelihood of HFpEF. The third and fourth step represent advanced workup with function testing and evaluation of etiology/phenotyping performed. We limited our analysis to the second step as this was a retrospective analysis in which the HFA-PEFF score was calculated after patient presentation, thus we cannot assume that any follow-on testing was performed due to the outcome of the test, particularly in those patients presenting prior to publication.

**Functional**

Septal and lateral mitral annular peak early diastolic velocity (e’)

- Major criterion: septal e’ < 7 cm/s or lateral e’ < 10 cm/s for age < 75 years
- Major criterion: septal e’ < 5 cm/s or lateral e’ < 7 cm/s for age ≥ 75 years

Average septal-lateral E/e’ ratio

- Major criterion: average septal-lateral E/e’ ≥ 15
- Minor criterion: average septal-lateral E/e’ 9-14

Tricuspid regurgitation peak velocity or pulmonary arterial systolic pressure

- Major criterion: TR peak velocity > 2.8 m/s
- Major criterion: Pulmonary artery systolic pressure > 35 mm Hg (calculated according to 4 x peak TR velocity plus estimated right atrial pressure)

Left ventricular global longitudinal systolic strain

- Major criterion: GLS < 16%

**Structural**

Left atrial volume indexed to body surface area

- Major criterion: > 34 mL/m2 in sinus rhythm
- Major criterion: > 40 mL/m2 in atrial fibrillation
- Minor criterion: 29-34 mL/m2 in sinus rhythm
- Minor criterion: 34-40 mL/m2 in atrial fibrillation

Left ventricular mass index and relative wall thickness

- Major criterion: LVMi ≥ 149 g/m2 in men or ≥ 122 g/m2 in women and RWT > 0.42
- Minor criterion: LVMi ≥ 115 g/m2 in men or ≥ 95 g/m2 in women or RWT > 0.42 or LV end-diastolic wall thickness ≥ 12 mm

**Biomarkers**

Natriuretic peptides

- Major criterion: NT-proBNP > 220 pg/mL or BNP > 80 pg/mL in sinus rhythm
- Major criterion: NT-proBNP > 660 pg/mL or BNP > 240 pg/mL in atrial fibrillation
- Minor criterion: NT-proBNP 125-220 pg/mL or BNP 35-80 pg/mL in sinus rhythm
- Minor criterion: NT-proBNP 375-660 pg/mL or BNP 105-240pg/mL in atrial fibrillation

**H2FPEF Score**

The continuous and categorical scores for H2FPEF was calculated according to Reddy et al.^9^. Specifically, for the categorical score, points were provided for criteria associated with patient’s body mass index, hypertension status, atrial fibrillation presence, pulmonary artery systolic pressure, age, and filling press estimate (see below). For the continuous score, the published coefficients for each of the categorical parameters (minus the hypertensive status) were used to generate a continuous predictor of HFpEF probability.

- Body mass index > 30 kg/m2 (2 point)
- 2 or more anti-hypertensive medicine (1 point)
- Paroxysmal or persistent atrial fibrillation (3 points)
- Doppler echocardiographic estimated pulmonary artery systolic pressure > 35 mm Hg (1 point)
- Age > 60 years (1 point)
- Doppler echocardiographic E/e’ > 9 (1 point)

For determination of anti-hypertensive medications, all patients with a diagnosis of hypertension on their medical records were assumed to be taking 2 or more medications. For the size of the training, validation, and independent testing cohort, it was not feasible to examine prescriptions for each patient. Thus, this might result in an overestimation of HFpEF probability for a patient with a hypertension diagnosis, albeit taking fewer than 2 medications.

# Results

Calibration plots are provided below for the AI HFpEF model training, validation, and independent testing dataset. In all instances, all data are retained for plots without considering uncertainty (expected entropy). For training and validation this is appropriate due to the post-hoc nature of threshold decision making for uncertainty. For independent testing, assessment of calibration and statistical tests did not differ meaningfully when only data below the uncertainty threshold were retained.


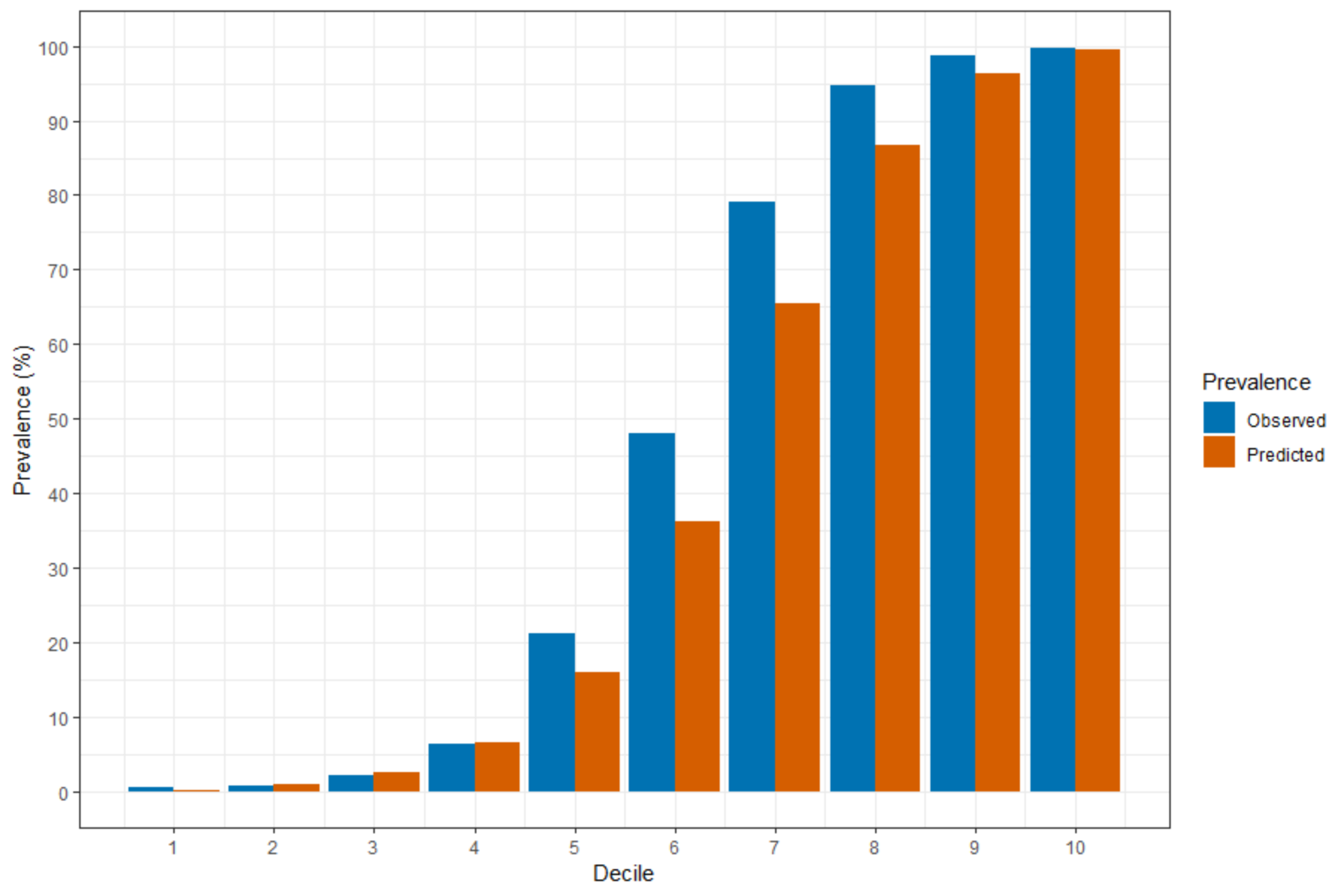


**Supplemental Figure 2**. Calibration plot for AI HFpEF model training dataset. Prediction class probability produced by the AI HFpEF model was separated into bins (n=10; x-axis) to ensure approximately equal number of predictions in each bin (n=622 or 623). In each bin, the observed (i.e., based on ground truth label), and predicted (based on AI HFpEF model) prevalence was calculated and plotted. The difference between the observed and predicted prevalence across all bins was used to calculate the Hosmer-Lemeshow test for goodness of fit (p<0.001). This test statistic indicates that the AI HFpEF model is not well calibrated in the training dataset, with “risk” underestimated in the middle deciles.


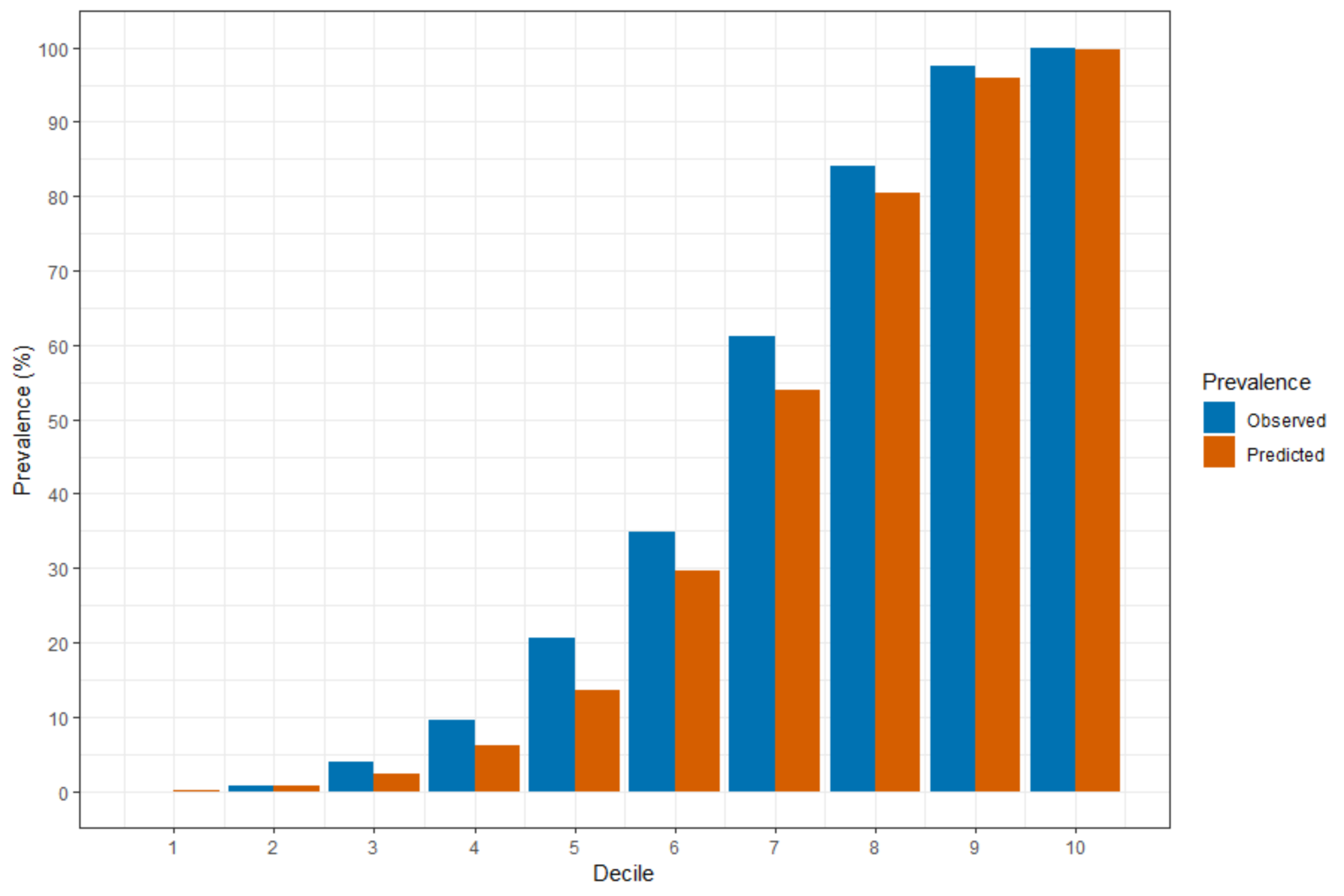


**Supplemental Figure 3**. Calibration plot for AI HFpEF model validation dataset. Prediction class probability produced by the AI HFpEF model was separated into bins (n=10; x-axis) to ensure approximately equal number of predictions in each bin (n=125 or 126). In each bin, the observed (i.e., based on ground truth label), and predicted (based on AI HFpEF model) prevalence was calculated and plotted. The difference between the observed and predicted prevalence across all bins was used to calculate the Hosmer-Lemeshow test for goodness of fit (p=0.049). This test statistic indicates that the AI HFpEF model is not well calibrated in the validation dataset, with “risk” underestimated in the middle deciles.


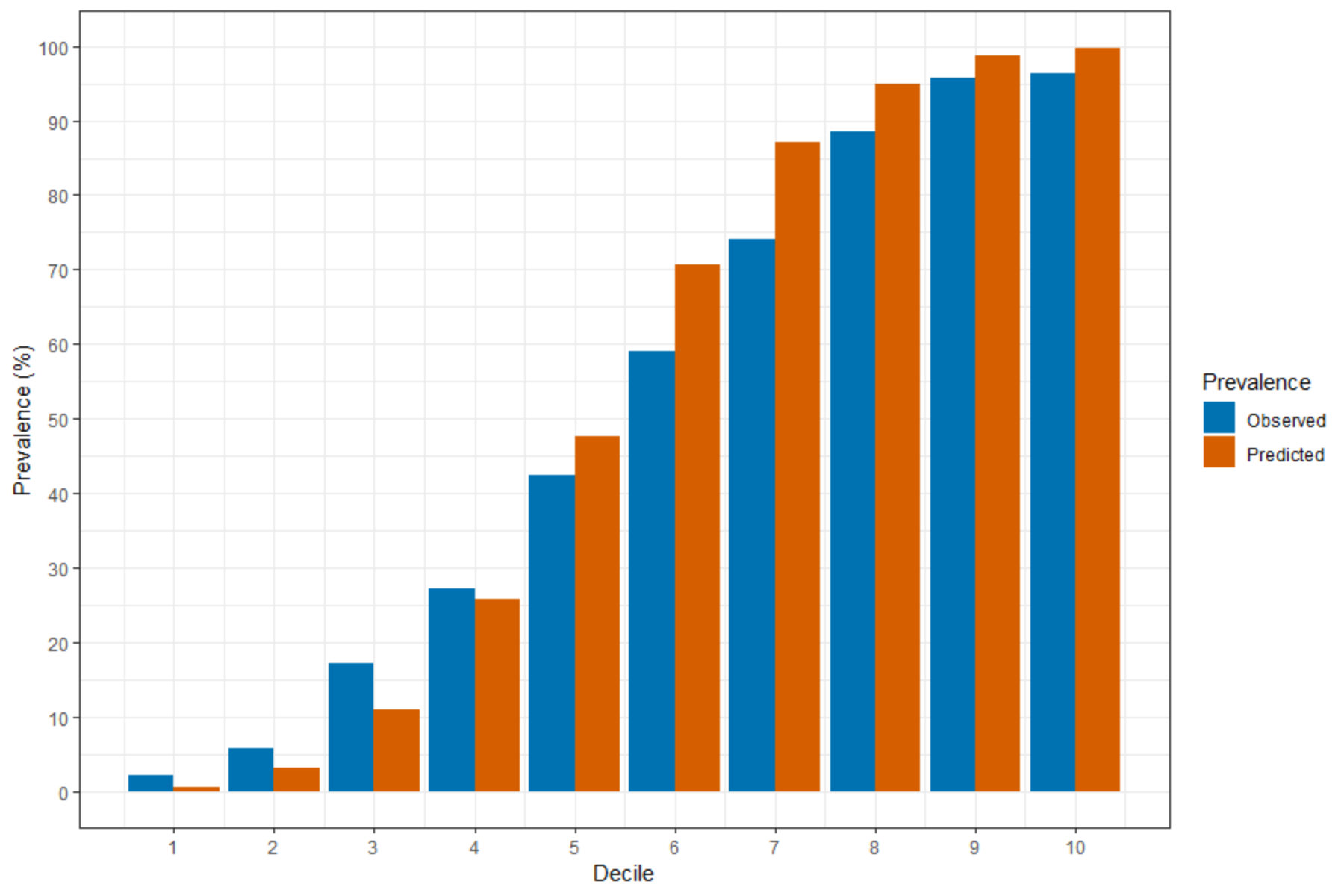


**Supplemental Figure 4**. Calibration plot for AI HFpEF model independent testing dataset. Prediction class probability produced by the AI HFpEF model was separated into bins (n=10; x-axis) to ensure approximately equal number of predictions in each bin (n=139 or 140). In each bin, the observed (i.e., based on ground truth label), and predicted (based on AI HFpEF model) prevalence was calculated and plotted. The difference between the observed and predicted prevalence across all bins was used to calculate the Hosmer-Lemeshow test for goodness of fit (p<0.001). This test statistic indicates that the AI HFpEF model is not well calibrated in the independent testing dataset, with “risk” overestimated in the middle deciles.

**Supplemental Table 2.** Patient descriptive statistics and demographics for key clinical trials in heart failure with preserved ejection fraction (HFpEF) and those used in training, validation, and independent testing of the AI HFpEF model

| Clinical Trial | EMPEROR-  PRESERVED | PARAGON-HF | TOPCAT | I-PRESERVE | CHARM-Preserved | PEP-CHF | DELIVER | Average | AI HFpEF model | | |
| --- | --- | --- | --- | --- | --- | --- | --- | --- | --- | --- | --- |
| Sample Size | 5988 | 4822 | 3445 | 4128 | 3023 | 850 | 6,263 | 28,519 | Training | Validation | Testing |
| Age (years) | 72 (9) | 73 (8) | 69 (10) | 72 (7) | 67 (11) | 75 (72–79) | 71.7 (9.6) | 71 (8) | 73 (12) | 74 (12) | 72 (13) |
| Women | 45 | 52 | 52 | 60 | 40 | 56 | 43.9 | 49.8 (40-60) | 50.5 | 53.7 | 52.2 |
| Obesity | 45 | 49 | 55 | 41 | 38 | NR | 77.7 | 51.0 (38-77.7) | 76.1 | 75.3 | 78.0 |
| White | 76 | 82 | 89 | 93 | 92 | N/A | 71.2 | 83.9 (71.2-93) | 89.1 | 87.3 | 44.4 |
| Black | 4 | 2 | 9 | 2 | 4 | N/A | 2.5 | 3.9 (2-9) | 1.9 | 1.4 | 37.9 |
| Other | 20 | 14 | 2 | 1 | 2 |  | 73.7 | 18.8 (1-73.7) | 1.7 | 1.8 | 0.5 |
| Hypertension | 90 | 96 | 91 | 89 | 64 | 79 | 88.7 | 85.4 (64-96) | 78.8 | 79.1 | 86.5 |
| Diabetes | 49 | 43 | 32 | 27 | 28 | 21 | 44.8 | 35.0 (21-49) | 39.6 | 38.8 | 50.5 |
| Chronic Kidney  Disease | 50 | 47 | 39 | 31 | 35 | NR | NR | 40.4 (31-50) | 24.7 | 24.7 | 46.8 |
| Coronary Artery  Disease | 35 | 43 | 59 | 13 | 33 | NR | 50.5 | 38.9 (13-59) | 33.6 | 31.1 | 31.6 |
| Atrial Fibrillation/  Atrial Flutter | 52 | 52 | 35 | 29 | 29 | NR | 56.7 | 42.3 (29-56.7) | 35.0 | 35.4 | 35.1 |
| Pulmonary Disease | 8.5 | 14 | 12 | NR | NR | NR | 9.4 | 11.0 (8.5-14) | 35.5 | 33.8 | 39.5 |
| Previous event | 19.5 | 16.5 | 17 | 17 | 26.5 | 27 | 12.5 | 19.4 (12.5-27) | 36.9 | 36.5 | 40.9 |

**Note**: Data are taken from Emperor-Preserved ^25^, Paragon-HF ^26^, TOPCAT ^27^, I-PRESERVE ^28^, CHARM-Preserved ^29^, PEP-CHF ^30^, and DELVER ^31^, and adapted from those presented in Anker et al. 2020 ^32^. Age in mean (SD) other than PEP-CHF which is median (IQR). Previous cardio- or cerebro-vascular event is taken as the average of myocardial infarction and stroke. Pulmonary disease taken as the average of obstructive/central sleep apnea and chronic obstructive pulmonary disorder. When averages for previous event and pulmonary disease were not possible due to NR or N/A for one of the levels, the only remaining value was taken as the estimate. For “Average” column, age refers to average mean and average SD from trials, whereas all other data are presented as average (range). HFpEF (cases) patients for current AI HFpEF model training, validation, and testing reported. N/A, not applicable; NR, not reported.

# Results: Grad-CAM

Figure 2 in the main manuscript represents a gradient-weighted class activation mapping (Grad-CAM) for a correctly classified patient and incorrectly classified patient (classified case, actual control; panel B). The patient in panel A (classified case, actual case) represents a 45-year-old female with structural heart disease (left ventricular mass index 143 g/m2, relative wall thickness 0.44, posterior wall thickness 12 mm) and echocardiographic evidence of significantly increased intra-cardiac filling pressure (septal e’ 4 cm/s, septal E/e’ 30, E/A ratio 3.0, mitral E deceleration time 182 ms, tricuspid regurgitation velocity 3.2 m/s). The AI HFpEF model provided an output with low uncertainty (expected entropy <0.001) and high prediction class probability (>0.999) resulting in a diagnostic positive (HFpEF) output. The patient in panel B (classified case, actual control) represents a 57-year-old female with borderline structural heart disease (left ventricular mass index 96 g/m2, relative wall thickness 0.41, posterior wall thickness 10 mm) but equivocal estimates of intracardiac filling pressure (septal e’ 4 cm/s, septal E/e’ 20, lateral e’, 7 cm/s, lateral E/e’ 11.4, E/A ratio 0.9, mitral E deceleration time 236 ms, tricuspid regurgitation velocity 2.2 m/s). The AI HFpEF model provided an output with higher uncertainty (expected entropy just below 0.59 threshold; 0.55) and prediction class probability above the classification threshold (0.62) resulting in a diagnostic positive (HFpEF) output.

# Results: Sub-group analyses

***Age, sex, and year of echocardiogram matched***

**Supplemental Table 3.** Analysis of AI HFpEF model classification performance when cases and controls are strictly matched for all permutations of the key matching factors (age, sex, and year of echocardiogram)

| Matching Criteria | tp | tn | fp | fn | Sensitivity (95% CI) | Specificity (95% CI) | Positive predictive value (95% CI) | Negative predictive value (95% CI) |
| --- | --- | --- | --- | --- | --- | --- | --- | --- |
| Age, Sex, Year | 217 | 207 | 52 | 42 | 83.8 (79.6, 87.6) | 79.9 (75.6, 84) | 80.7 (76.6, 84.8) | 83.1 (79.3, 86.9) |
| Age, Sex | 378 | 340 | 93 | 55 | 87.3 (84.7, 90) | 78.5 (75.2, 81.7) | 80.3 (76.9, 83.4) | 86.1 (83.2, 89.0) |
| Age, Year | 259 | 241 | 63 | 45 | 85.2 (81.7, 88.6) | 79.3 (75.4, 82.9) | 80.4 (76.7, 84) | 84.3 (80.7, 87.8) |
| Year, Sex | 476 | 448 | 97 | 69 | 87.3 (85, 89.6) | 82.2 (79.4, 85) | 83.1 (80.2, 85.8) | 86.7 (84.1, 89) |
| Age | 388 | 354 | 92 | 58 | 87 (84.3, 89.5) | 79.4 (76.1, 82.9) | 80.8 (77.8, 83.9) | 85.9 (83.1, 88.8) |
| Sex | 510 | 477 | 105 | 72 | 87.6 (85.1, 89.8) | 82 (79.3, 84.6) | 82.9 (80.4, 85.3) | 86.9 (84.3, 89.2) |
| Year | 485 | 455 | 101 | 71 | 87.2 (84.4, 89.6) | 81.8 (78.9, 84.6) | 82.8 (80.1, 85.4) | 86.5 (83.8, 89) |

**Note**: tp, true positive; tn, true negative; fp, false positive; fn, false negative; 95% CI, 95% confidence interval

***Prevalence of disease***


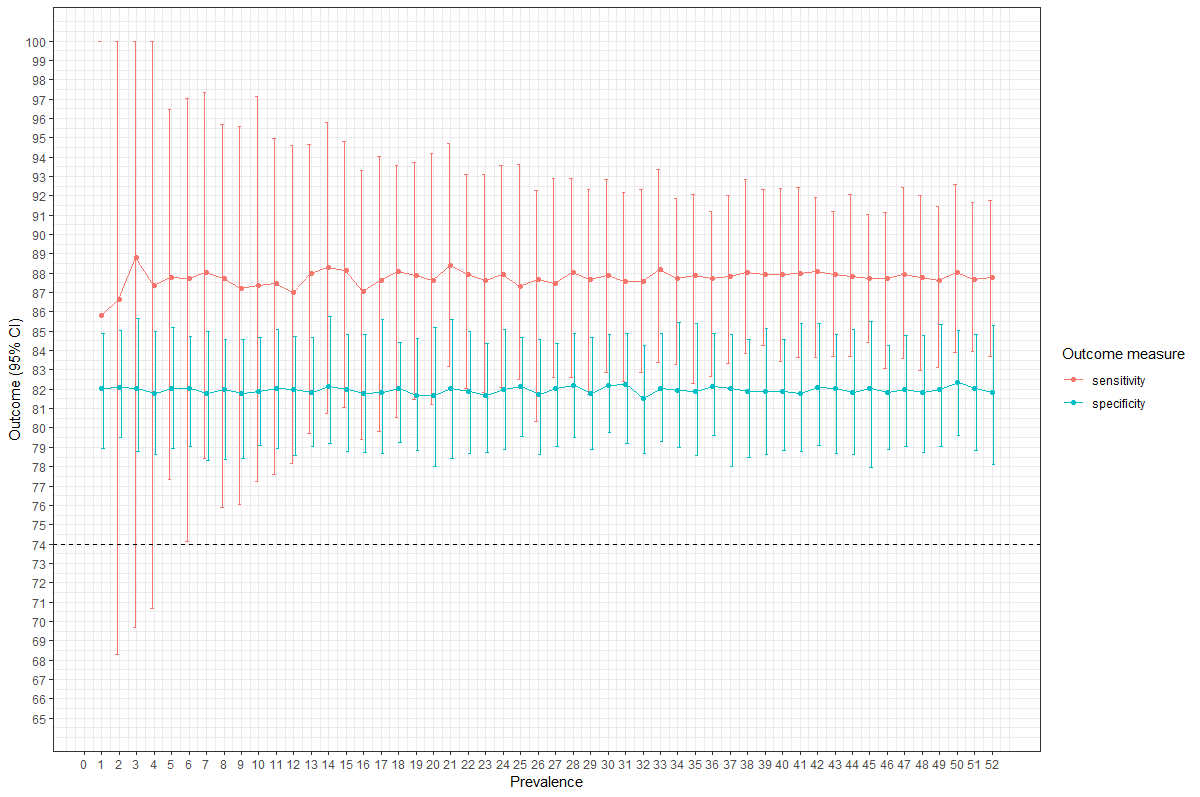


**Supplemental Figure 5.** Analysis of AI HFpEF model classification performance (sensitivity and specificity) when prevalence of disease ranges from 1% to ~52%, representing a very conservative to fully enriched population.


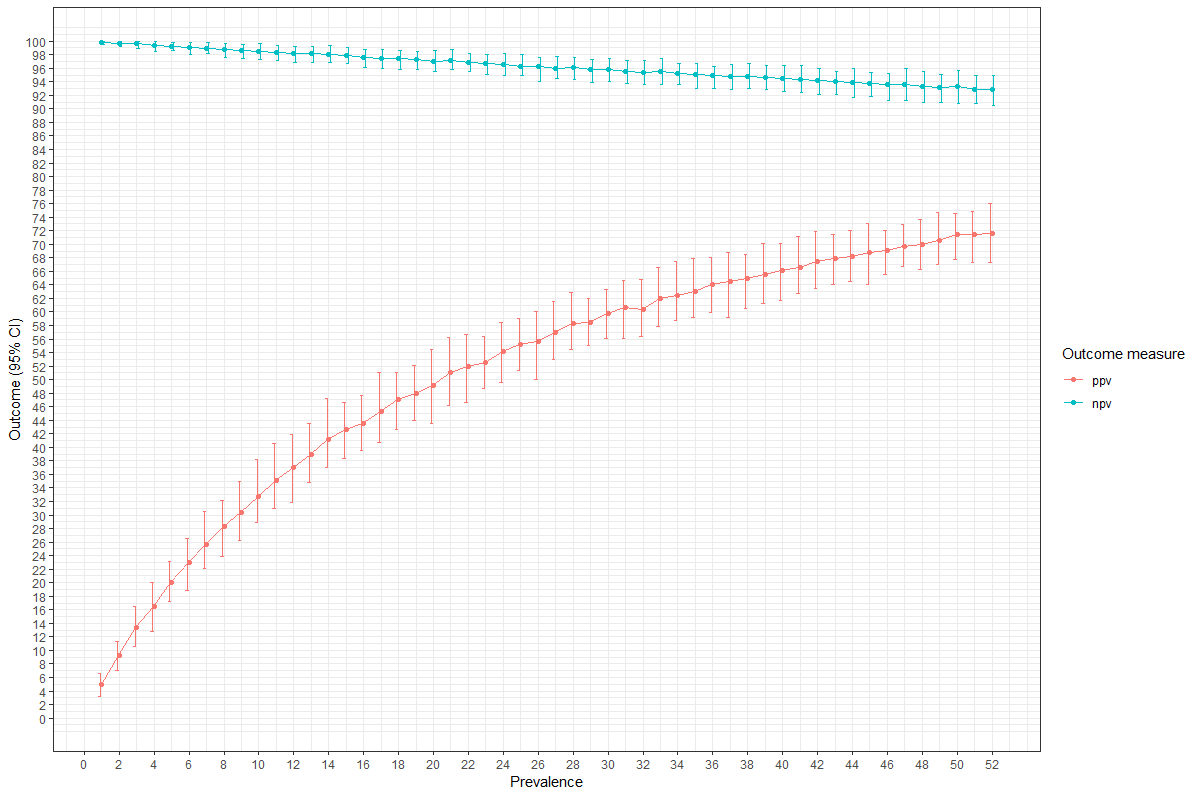


**Supplemental Figure 6.** Analysis of AI HFpEF model classification performance (positive and negative predictive value) when prevalence of disease ranges from 1% to ~52%, representing a very conservative to fully enriched population.

***Subgroup analyses***

**Supplemental Table 4.** Analysis of AI HFpEF model classification performance in key sub-groups of interest

| Descriptor | tp | tn | fp | fn | Sensitivity  (95% CI) | Specificity  (95% CI) | Positive predictive value  (95% CI) | Negative predictive value  (95% CI) |
| --- | --- | --- | --- | --- | --- | --- | --- | --- |
| **Procedure Year** |  |  |  |  |  |  |  |  |
| 2009 | 24 | 24 | 8 | 5 | 82.8 (67.3, 100) | 75 (56.2, 90.0) | 82.8 (64.5, 100) | 75 (58.8, 89.2) |
| 2010 | 27 | 23 | 7 | 6 | 81.8 (62.7, 94.2) | 76.7 (60, 96.3) | 79.3 (57.7, 93.5) | 79.4 (64.7, 94.7) |
| 2011 | 28 | 30 | 10 | 9 | 75.7 (59.6, 87.5) | 75 (57.8, 88.1) | 76.9 (62.5, 90.2) | 73.7 (54.8, 90.0) |
| 2012 | 52 | 38 | 6 | 7 | 88.1 (77.5, 95.1) | 86.4 (73.3, 95.4) | 84.4 (71.0, 93.0) | 89.7 (80.2, 96.7) |
| 2013 | 38 | 28 | 4 | 4 | 90.5 (78.5, 98.8) | 87.5 (69.6, 98.5) | 87.5 (72.0, 98.4) | 90.5 (78.0, 98.6) |
| 2014 | 39 | 31 | 11 | 3 | 92.9 (85.0, 100) | 73.8 (60.5, 88.1) | 91.2 (80.4, 100) | 78 (65, 90.41) |
| 2015 | 45 | 46 | 8 | 4 | 91.8 (82.6, 97.6) | 85.2 (74.0, 92.6) | 92 (83.7, 97.7) | 84.9 (74.8, 92.8) |
| 2016 | 46 | 38 | 16 | 5 | 90.2 (80, 97.5) | 70.4 (60.2, 85.7) | 88.4 (79.0, 97.5) | 74.2 (62.5, 87.2) |
| 2017 | 41 | 42 | 9 | 6 | 87.2 (75.8, 96.8) | 82.4 (69.9, 92.1) | 87.5 (76.6, 96.5) | 82 (71.9, 91.7) |
| 2018 | 37 | 36 | 6 | 0 | 100 (100, 100) | 85.7 (74.6, 96.5) | 100 (100, 100) | 86 (69.9, 96.8) |
| 2019 | 56 | 60 | 8 | 9 | 86.2 (76.7, 96.0) | 88.2 (80, 95.9) | 87 (79.2, 96.3) | 87.5 (79.6, 96.1) |
| 2020 | 84 | 68 | 11 | 13 | 86.6 (79.6, 94.4) | 86.1 (77.5, 93.9) | 84 (75.9, 92.4) | 88.4 (81.7, 94.9) |
| 2021 | 18 | 13 | 1 | 3 | 85.7 (63.0, 100) | 92.9 (75, 100) | 81.2 (57.2, 100) | 94.7 (78.2, 100) |
| **Site** |  |  |  |  |  |  |  |  |
| Albert Lea | 14 | 20 | 6 | 1 | 93.3 (76.9, 100) | 76.9 (55.6, 91.1) | 95.2 (84.8, 100) | 70 (45.2, 88.6) |
| Austin | 28 | 34 | 14 | 5 | 84.8 (68.3, 100) | 70.8 (57.9, 83.3) | 87.2 (72.2, 100) | 66.7 (48.4, 82.3) |
| Jacksonville | 157 | 187 | 33 | 27 | 85.3 (80.8, 91.1) | 85 (78.8, 90.1) | 87.4 (81.9, 91.9) | 82.6 (77.5, 87.7) |
| La Crosse | 46 | 39 | 8 | 10 | 82.1 (71.2, 92.0) | 83 (69.5, 93.7) | 79.6 (68.4, 91.2) | 85.2 (72.9, 95.2) |
| Mankato | 93 | 45 | 12 | 12 | 88.6 (81.1, 93.7) | 78.9 (66.2, 88.3) | 78.9 (67.0, 87.1) | 88.6 (81.5, 95.1) |
| Outreach | 70 | 60 | 23 | 7 | 90.9 (84.2, 98.1) | 72.3 (60.4, 79.7) | 89.6 (82.1, 97.75) | 75.3 (64.1, 84.6) |
| Red Wing | 4 | 5 | 2 | 0 | 100 (100, 100) | 71.4 (25, 85.7) | 100 (100, 100) | 66.7 (25, 83.4) |
| Scottsdale | 123 | 87 | 7 | 12 | 91.1 (82.8, 96.1) | 92.6 (88.1, 97.1) | 87.9 (78.0, 94.3) | 94.6 (91.1, 98.0) |
| **Sex** |  |  |  |  |  |  |  |  |
| Female | 279 | 241 | 52 | 37 | 88.3 (84.1, 92.0) | 82.3 (77.8, 87.7) | 86.7 (82.1, 90.9) | 84.3 (80.5, 88.5) |
| Male | 256 | 236 | 53 | 37 | 87.4 (82.6, 90.5) | 81.7 (77.8, 86.6) | 86.4 (81.3, 89.7) | 82.8 (78.9, 87.3) |
| **Age class** |  |  |  |  |  |  |  |  |
| <25 years | 0 | 10 | 0 | 0 | 0 | 100 | 0 | 100 |
| 25-54 years | 44 | 142 | 9 | 15 | 74.6 (60.2, 84.8) | 94 (88.7, 98.0) | 90.4 (83.7, 94.5) | 83 (69.2, 92.0) |
| 55-64 years | 90 | 108 | 12 | 17 | 84.1 (75.5, 91.9) | 90 (84.6, 95.1) | 86.4 (79.8, 93.0) | 88.2 (80.8, 94.4) |
| >=65 years | 401 | 217 | 84 | 42 | 90.5 (87.2, 93.1) | 72.1 (66.6, 77.6) | 83.8 (78.7, 88.3) | 82.7 (79.2, 86.1) |
| **Ethnicity/Race** |  |  |  |  |  |  |  |  |
| African American | 201 | 210 | 20 | 26 | 88.5 (83.6, 93.1) | 91.3 (87.7, 94.8) | 89 (83.0, 93.7) | 91 (86.6, 94.5) |
| Caucasian | 249 | 212 | 47 | 30 | 89.2 (84.5, 93.5) | 81.9 (76.8, 86.8) | 87.6 (82.4, 92.4) | 84.1 (79.1, 88.7) |
| Other | 3 | 4 | 0 | 0 | 100 | 100 | 100 | 100 |
| **Obesity** |  |  |  |  |  |  |  |  |
| Absent | 111 | 152 | 24 | 22 | 83.5 (76.6, 90.1) | 86.4 (79.2, 92.0) | 87.4 (81.1, 92.7) | 82.2 (73.2, 88.6) |
| Present | 424 | 325 | 80 | 52 | 89.1 (86.3, 91.9) | 80.2 (76.2, 84.2) | 86.2 (82.5, 89.5) | 84.1 (81.1, 87.3) |
| **Hypertension** |  |  |  |  |  |  |  |  |
| Absent | 71 | 285 | 35 | 10 | 87.7 (79.2, 96.0) | 89.1 (85.4, 92.9) | 96.6 (93.6, 98.9) | 67 (53.6, 76.9) |
| Present | 464 | 192 | 70 | 64 | 87.9 (85.3, 90.8) | 73.3 (66.6, 79.3) | 75 (69.1, 81.1) | 86.9 (83.5, 90.1) |
| **Hyperlipidemia** |  |  |  |  |  |  |  |  |
| Absent | 143 | 250 | 33 | 24 | 85.6 (80, 90.7) | 88.3 (84.4, 92.3) | 91.2 (87.6, 94.7) | 81.2 (73.1, 87.7) |
| Present | 392 | 227 | 72 | 50 | 88.7 (85.4, 91.9) | 75.9 (69.3, 80.6) | 81.9 (76.5, 86.3) | 84.5 (80.4, 87.6) |
| **Structural heart disease** |  |  |  |  |  |  |  |  |
| Absent | 59 | 221 | 28 | 16 | 78.7 (66.1, 87.2) | 88.8 (85.0, 93.6) | 93.2 (89.8, 96.7) | 67.8 (57.6, 78.7) |
| Present | 476 | 256 | 77 | 58 | 89.1 (85.1, 91.9) | 76.9 (71, 82.4) | 81.5 (76.5, 86.0) | 86.1 (82.8, 90.5) |
| **Atrial Fibrillation** |  |  |  |  |  |  |  |  |
| Absent | 332 | 421 | 82 | 59 | 84.9 (80.4, 88.8) | 83.7 (80.0, 86.9) | 87.7 (84.5, 90.7) | 80.2 (76.4, 85.2) |
| Present | 203 | 56 | 23 | 15 | 93.1 (88.1, 96.3) | 70.9 (58.0, 86.6) | 78.9 (66.0, 89.5) | 89.8 (84.8, 95.0) |
| **Coronary Artery Disease** |  |  |  |  |  |  |  |  |
| Absent | 352 | 434 | 88 | 62 | 85 (80.9, 88.8) | 83.1 (79.0, 86.8) | 87.5 (84.1, 90.4) | 80 (75.5, 84.1) |
| Present | 183 | 43 | 17 | 12 | 93.8 (90.0, 97.7) | 71.7 (57.9, 83.5) | 78.2 (64.4, 89.8) | 91.5 (86.9, 96.0) |
| **Chronic Kidney Disease** |  |  |  |  |  |  |  |  |
| Absent | 275 | 421 | 94 | 46 | 85.7 (80.2, 89.0) | 81.7 (77.3, 85.7) | 90.1 (87.0, 93.0) | 74.5 (67.7, 79.5) |
| Present | 260 | 56 | 11 | 28 | 90.3 (85.5, 93.7) | 83.6 (72.6, 93.2) | 66.7 (55.6, 77.4) | 95.9 (92.9, 98.4) |
| **Diabetes Mellitus** |  |  |  |  |  |  |  |  |
| Absent | 262 | 408 | 83 | 41 | 86.5 (82.5, 90.7) | 83.1 (78.9, 87.7) | 90.9 (88.0, 93.7) | 75.9 (70.8, 81.9) |
| Present | 273 | 69 | 22 | 33 | 89.2 (85.5, 92.6) | 75.8 (63.8, 83.5) | 67.6 (57.5, 78.1) | 92.5 (87.9, 94.8) |
| **Pulmonary disease** |  |  |  |  |  |  |  |  |
| Absent | 318 | 402 | 84 | 49 | 86.6 (82.0, 90.9) | 82.7 (78.4, 86.7) | 89.1 (85.2, 92.5) | 79.1 (75.0, 83.5) |
| Present | 217 | 75 | 21 | 25 | 89.7 (85.2, 94.0) | 78.1 (67.4, 85.7) | 75 (64.8, 84.9) | 91.2 (86.3, 94.6) |
| **Previous cardio- or**  **cerebro-vascular event** |  |  |  |  |  |  |  |  |
| Absent | 306 | 397 | 78 | 52 | 85.5 (81.2, 88.6) | 83.6 (79.7, 87.6) | 88.4 (85.5, 90.9) | 79.7 (75.6, 84.3) |
| Present | 229 | 80 | 27 | 22 | 91.2 (86.5, 94.8) | 74.8 (64.4, 83.2) | 78.4 (67.3, 86.3) | 89.5 (84.3, 93.1) |
| **Manufacturer** |  |  |  |  |  |  |  |  |
| GE Healthcare | 375 | 338 | 70 | 47 | 88.9 (85.3, 91.5) | 82.8 (79.6, 88.2) | 87.8 (83.9, 91.2) | 84.3 (80.7, 89.2) |
| Siemens | 26 | 36 | 5 | 10 | 72.2 (55.8, 87.1) | 87.8 (75.8, 97.0) | 78.3 (66.7, 89.1) | 83.9 (68.6, 95.9) |
| Philips Medical Systems | 134 | 103 | 30 | 17 | 88.7 (83.6, 93.1) | 77.4 (68.5, 84.9) | 85.8 (80.2, 90.8) | 81.7 (74.1, 86.6) |
| **Frames/Cardiac cycle** |  |  |  |  |  |  |  |  |
| <30 | 362 | 378 | 68 | 60 | 85.8 (81.2, 89.4) | 84.8 (81.0, 88.2) | 86.3 (81.5, 89.7) | 84.2 (79.5, 87.8) |
| >=30 | 160 | 95 | 33 | 12 | 93 (88.3, 96.5) | 74.2 (66.7, 80.3) | 88.8 (81.2, 94.2) | 82.9 (77.9, 87.7) |
| **Quality of image analysed** |  |  |  |  |  |  |  |  |
| **Is ectopy or irregular rhythm present during acquisition?** |  |  |  |  |  |  |  |  |
| No | 271 | 267 | 39 | 37 | 88.0 (84.4, 92.1) | 87.3 (83.6, 91.0) | 87.4 (83.6, 91.3) | 87.8 (83.8, 91.9) |
| Yes | 12 | 9 | 1 | 3 | 80.0 (56.2, 100) | 90 (61.4, 100) | 92.3 (66.7, 100) | 75 (43.5, 100) |
| **4 Chamber View Overall Quality*** |  |  |  |  |  |  |  |  |
| Poor | 40 | 30 | 13 | 6 | 87.0 (74.2, 96.8) | 69.8 (53.7, 83.1) | 75.5 (59.0, 85.6) | 83.3 (67.5, 95.9) |
| Fair | 83 | 80 | 8 | 10 | 89.2 (81.3, 96.7) | 90.9 (85.6, 96.4) | 91.2 (84.7, 96.1) | 88.9 (83.3, 96.9) |
| Good | 160 | 166 | 19 | 24 | 87 (80.6, 91.2) | 89.7 (83.6, 94.3) | 89.4 (84.3, 93.9) | 87.4 (81.7, 91.1) |
| **4 Chamber Gain** |  |  |  |  |  |  |  |  |
| Poor | 44 | 35 | 10 | 8 | 84.6 (75.7, 92.6) | 77.8 (64.3, 91.3) | 81.5 (68.8, 91.9) | 81.4 (70.2, 90.7) |
| Fair | 156 | 196 | 22 | 22 | 87.6 (81.9, 92.1) | 89.9 (83.5, 94.4) | 87.6 (81.6, 93.5) | 89.9 (85.6, 93.8) |
| Optimal | 83 | 45 | 8 | 10 | 89.2 (79.6, 95.0) | 84.9 (75, 93.6) | 91.2 (84.7, 97.0) | 81.8 (66.7, 91.0) |
| **4 Chamber endocardial border definition** |  |  |  |  |  |  |  |  |
| 3 or more segments not seen | 43 | 36 | 8 | 7 | 86 (75.0, 95.0) | 81.8 (65.2, 93.3) | 84.3 (71.2, 94.0) | 83.7 (75, 94.8) |
| 1 or 2 segments not seen | 63 | 87 | 13 | 10 | 86.3 (76.0, 92.4) | 87 (79.6, 93.1) | 82.9 (73.0, 91.2) | 89.7 (81.1, 94.4) |
| All segments visualised | 177 | 153 | 19 | 23 | 88.5 (83.2, 93.5) | 89 (84.8, 94.8) | 90.3 (86.3, 95.1) | 86.9 (81.4, 92.4) |

**Note**: tp, true positive; tn, true negative; fp, false positive; fn, false negative; 95% CI, 95% confidence interval. 95% confidence intervals not provided when assessing only true positive or true negatives (i.e., 100%).* Refers to true view, not foreshortened, over or under-rotated, and all 4 chambers visible, the LV apex is cantered, and the septum nearly vertical, deviating less than 30° from vertical, and both the mitral and tricuspid valve are visible.

# Results: Clinical Utility of AI HFpEF Model

***Clinical utility of AI HFpEF model***


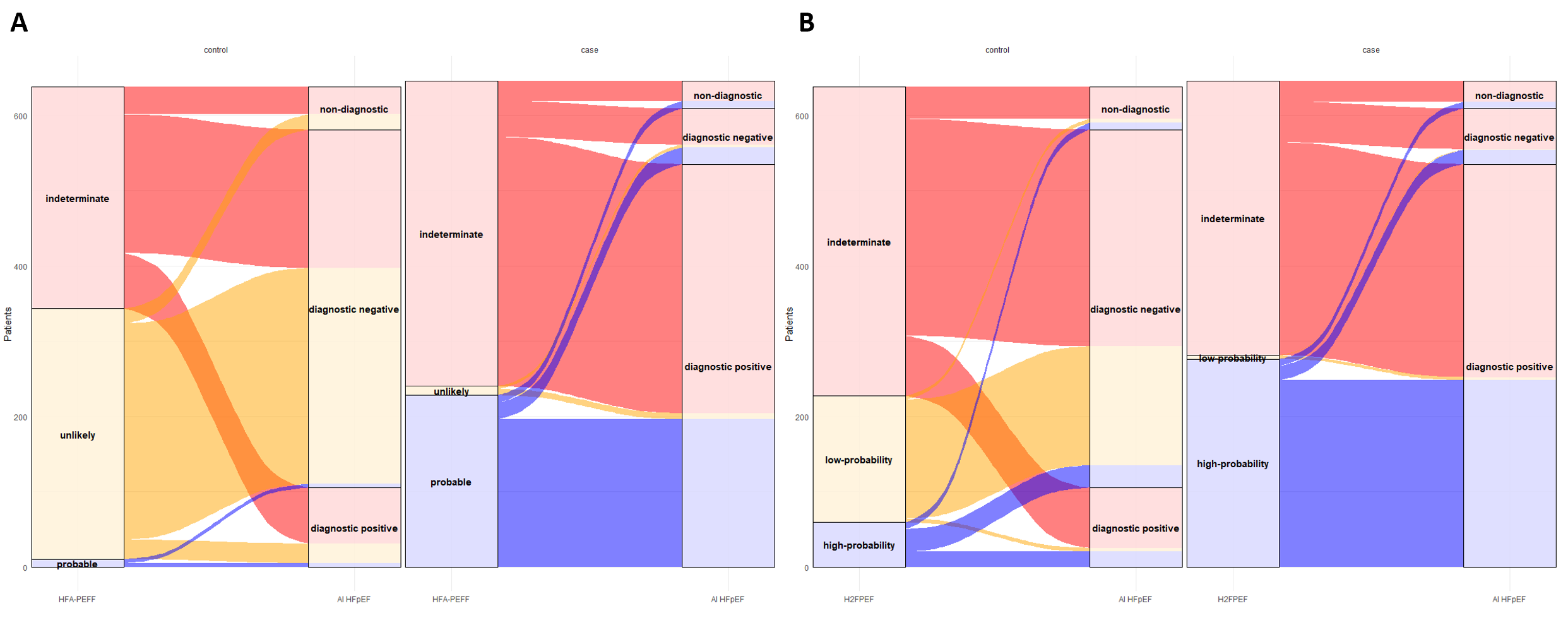


**Supplemental Figure 7**. Alluvial plot demonstrating reclassification of patients using the HFA-PEFF score (panel A) and H2FPEF score (panel B) compared to the AI HFpEF model, separated into controls (left side) and cases (right side).

**Decision Curve Analysis**

The decision curves presented in the main manuscript, representing the number of cases identified and patient management decisions utilizing the AI HFpEF model, should be interpreted as follows.

Figures represent decision curve analysis comparing net benefit of patient management decisions are based on the output of a clinically validated algorithm (HFA-PEFF, panel A; H2FPEF, panel B) and/or the AI HFpEF model. In the scenarios presented, the management/treatment decision is assumed to represent prescription of SGLT2i to the patient. The red and gold lines represent clinical baselines of managing all patients (red) or no patients (gold), regardless of the output of any test. The baseline clinical approach utilizing empirical tests is represented in green. In this scenario, any patient with a probable likelihood of HFpEF according to the HFA-PEFF or H2FPEF scores would be prescribed SGLT2i. The blue line represents a joint approach, managing patients based on either a probable output from the HFA-PEFF or H2FPEF score, or a positive diagnostic output from the AI HFpEF model (i.e., “HFpEF”). The purple line represents a conditional approach, wherein patients are managed based on either a probable likelihood of HFpEF according to the HFA-PEFF or H2FPEF scores or indeterminate likelihood according to these score plus a positive diagnostic output from the AI HFpEF model. The x axis refers to the threshold probability that would be required by a clinician and/or patient to initiate a given management/treatment. This threshold probability likely differs in various clinical scenarios, but simply, it represents the relative harm of avoiding a given management/treatment option for a patient with the disease, compared to unnecessarily intervening on a patient who is disease free. In the context of the current study, the minimum threshold probability one might expect would be 30%. This represents the trade-off between the risk of an adverse event when taking SGLT2i (5.8%; DELIVER study), and the risk reduction associated with taking an SGLT2i (-19% for HF hospitalization or worsening HF event; DELIVER study). The y-axis refers to the net benefit of taking a given approach, with units presenting the number of true positives identified per 100 in the target population (e.g., 0.10 represents 10 patients per 100). Thus, assuming a 20% prevalence of HFpEF in the target population (cardiology/heart failure clinic), and a threshold probability of 30%, managing a patient based on the combined information provided by the HFA-PEFF or H2FPEF score and the AI HFpEF model (blue or purple) results in 5 to 6 more patients with HFpEF (out of assumed 20 in the population) being managed appropriately compared to managing based on the HFA-PEFF or H2FPEF scores alone.

Decision curves representing the impact on net reduction in interventions following a similar paradigm are highlighted below.


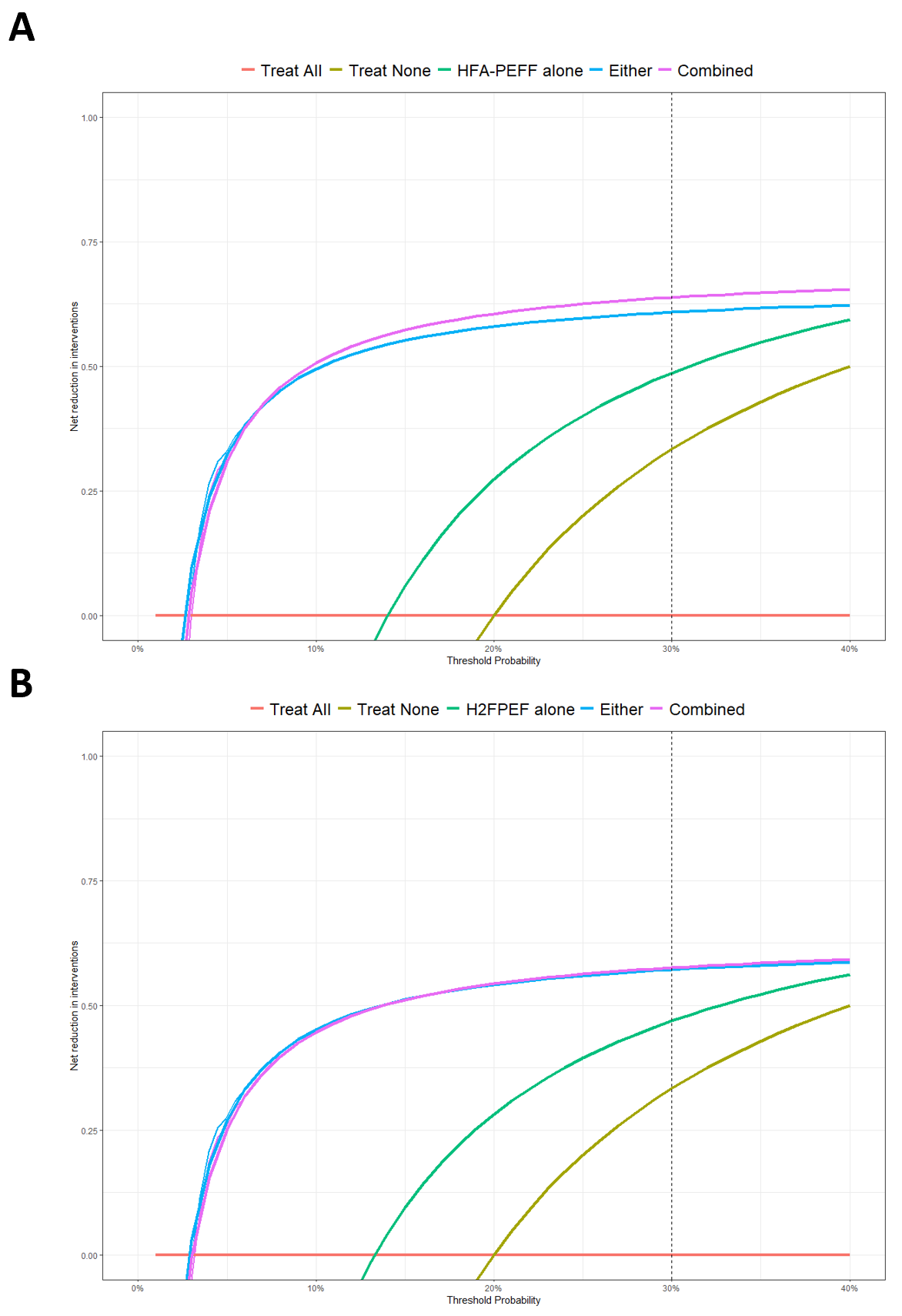


**Supplemental Figure 8**. Decision curve analysis comparing net reduction in interventions when patient management decisions are based on the output of a clinically validated algorithm (HFA-PEFF, panel A; H2FPEF, panel B) and/or the AI HFpEF model. In the scenarios presented, the management/treatment decision is assumed to represent prescription of SGLT2i to the patient. The red and gold lines represent clinical baselines of managing all patients (red) or no patients (gold), regardless of the output of any test. The baseline clinical approach utilizing empirical tests is represented in green. In this scenario, any patient with a probable likelihood of HFpEF according to the HFA-PEFF or H2FPEF scores would be prescribed SGLT2i. The blue line represents a joint approach, managing patients based on either a probable output from the HFA-PEFF or H2FPEF score, or a positive diagnostic output from the AI HFpEF model (i.e., “HFpEF”). The purple line represents a conditional approach, wherein patients are managed based on either a probable likelihood of HFpEF according to the HFA-PEFF or H2FPEF scores or indeterminate likelihood according to these score plus a positive diagnostic output from the AI HFpEF model. The x axis refers to the threshold probability that would be required by a clinician and/or patient to initiate a given management/treatment. This threshold probability likely differs in various clinical scenarios, but simply, it represents the relative harm of avoiding a given management/treatment option for a patient with the disease, compared to unnecessarily intervening on a patient who is disease free. In the context of the current study, the minimum threshold probability one might expect would be 30%. This represents the trade-off between the risk of an adverse event when taking SGLT2i (5.8%; DELIVER study), and the risk reduction associated with taking an SGLT2i (-19% for HF hospitalization or worsening HF event; DELIVER study). The y-axis refers to the net reduction in unnecessary interventions/management decision with units presenting the number of true negatives identified per 100 in the target population (e.g., 0.10 represents 10 patients per 100). Thus, assuming a 20% prevalence of HFpEF in the target population (cardiology/heart failure clinic), and a threshold probability of 30%, managing a patient based on the combined information provided by the HFA-PEFF or H2FPEF score and the AI HFpEF model (blue or purple) results in approximately 11 to 15 more patients without HFpEF being managed appropriately (out of assumed 80 in population) compared to managing based on the HFA-PEFF or H2FPEF scores alone.

# Results: Clinical Endpoints

There were 22 patients in the test group (age 69± 10 years, 15 (68%) women), with right heart catheterization within 1 year (median (IQR): 45 (7-161) days) of the echocardiogram. These included 19 patients in the HF group and 3 controls. Of the patients in the HF group, the diagnosis of HFpEF (pulmonary capillary wedge pressure ≥15 mmHg at rest) was confirmed in 16. All were classified as HFpEF by AI (sensitivity of algorithm 100%, 16/16). HFpEF was not present by catheterization in 3 although all were positive by the classifier; exercise hemodynamics were not assessed at the time of catheterization. In one patient with wedge pressure 13 mm Hg, mean pulmonary artery (PA) pressure was mildly elevated (24-28 mm Hg) and endomyocardial biopsy revealed wild type ATTR. In another, the diagnosis by catheterization was diffuse coronary disease, s/p 4 vessel bypass and occluded vein graft, and pulmonary hypertension (wedge pressure 13 mm Hg, mean PA pressure 27 mm Hg). One had mild coronary artery disease and pulmonary hypertension, wedge pressure 6 mm Hg and mean PA pressure 32 mm Hg.

Of the three patients in the control group, none had HFpEF by catheterization (severe coronary artery disease in 2 and pulmonary hypertension in 1); 2 were negative for HFpEF by AI (specificity 67%, 2/3). The false positive by AI had prior 5- vessel coronary artery bypass grafting, severe coronary artery disease, and normal PA and wedge pressures; echocardiographic measurements included E/e’ 11.7, normal left atrial size and RV systolic pressure, left ventricular ejection fraction 67%, moderately increased left ventricular mass index and mildly reduced right ventricular systolic function. His dyspnea was attributed to coronary artery disease and hypertensive heart disease without heart failure.

**Supplementary Table 5.** PRIME Checklist ^33^

| Section | Checklist item |  |
| --- | --- | --- |
| **1** | **Designing the study plan** |  |
| 1.1 | Describe the need for the application of machine learning to the dataset | Pg 7- 8 (introduction) |
| 1.2 | Describe the objectives of the machine learning analysis | Pg 7 - 8 (introduction) |
| 1.3 | Define the study plan | Pg 8 (introduction), pg 9 (methods), Figure 1 |
| 1.4 | Describe the summary statistics of baseline data | Pg 16 (results), Table 1 |
| 1.5 | Describe the overall steps of machine learning workflow | Pg 11-13 (methods), Central Illustration |
| **2** | **Data standardization, feature engineering, and learning** |  |
| 2.1 | Describe how the data were processed in order to make it clean, uniform, and consistent | Pg 11-13 (methods) |
| 2.2 | Describe whether variables were normalized and if so, how this was done | Pg 11-13 (methods) |
| 2.3 | Provide details on the fraction of missing values (if any) and imputation methods | Table 1 |
| 2.4 | Perform and describe feature selection process | NA |
| 2.5 | Identify and describe the process to handle outliers if any | NA |
| 2.6 | Describe whether class imbalance existed, and which method was applied to deal with it | NA |
| **3** | **Selection of Machine Learning Model** |  |
| 3.1 | Explicitly define the goal of the analysis e.g., regression, classification, clustering | Pg 11-13 (methods) |
| 3.2 | Identify the proper learning method used (e.g., supervised, reinforcement learning etc.) to address the problem | Pg 11-13 (methods) |
| 3.3 | Provide explicit details on the use of simpler, complex, or ensemble models | Pg 11-13 (methods) |
| 3.4 | Provide the comparison of complex models against simpler models if possible | Table 4 |
| 3.5 | Define ensemble methods, if used | NA |
| 3.6 | Provide details on whether the model is interpretable | Pg 14 (methods), pg 16 (results), pg 21-22 (discussion), Table 2, Figure 2 |
| **4** | **Model Assessment** |  |
| 4.1 | Provide a clear description of data used for training, validation, and testing | Pg 16 (results), Table 1 |
| 4.2 | Describe how the model parameters were optimized (e.g., optimization technique, number of model parameters etc.) | Pg 11-13 (methods) |
| **5** | **Model Evaluation** |  |
| 5.1 | Provide the metric(s) used to evaluate the performance of the model | Pg14-15 (methods), Pg 16-19 (results) |
| 5.2 | Define the prevalence of disease and the choice of the scoring rule used | Pg 9-10 (methods), Supplement Pg 11, Supplement Figure 5, Supplement Figure 6 |
| 5.3 | Report any methods used to balance the numbers of subjects in each class | Supplement Pg 11, Supplement Table 3 |
| 5.4 | Discuss the risk associated to misclassification | Pg 16 (results), Pg 20-22 (discussion) |
| **6** | **Best Practices for Model Replicability** |  |
| 6.1 | Consider sharing code or scripts on public repository with appropriate copyright protection steps for further development and non-commercial use | NA |
| 6.2 | Release data dictionary with appropriate explanation of the variables | NA |
| 6.3 | Document version of all software and external libraries | Pg 15 methods, Supplemental References |
| **7** | **Reporting limitations, biases and alternatives** |  |
| 7.1 | Identify and report the relevant model assumptions and findings | Results |
| 7.2 | If well performing models were tested on a hold-out validation dataset, detail the data of that validation set with the same rigor as that of training dataset (see section 2 above) | Table 1 |

# References

1 Mitchell C, Rahko PS, Blauwet LA, *et al.* Guidelines for Performing a Comprehensive Transthoracic Echocardiographic Examination in Adults: Recommendations from the American Society of Echocardiography. *Journal of the American Society of Echocardiography* 2019; **32**: 1–64.

2 Lang RM, Badano LP, Mor-Avi V, *et al.* Recommendations for Cardiac Chamber Quantification by Echocardiography in Adults: An Update from the American Society of Echocardiography and the European Association of Cardiovascular Imaging. *Eur Heart J Cardiovasc Imaging* 2015; **16**: 233–71.

3 Heidenreich PA, Bozkurt B, Aguilar D, *et al.* 2022 AHA/ACC/HFSA Guideline for the Management of Heart Failure: A Report of the American College of Cardiology/American Heart Association Joint Committee on Clinical Practice Guidelines. *Circulation* 2022; **145**. DOI:10.1161/CIR.0000000000001063.

4 Nagueh SF, Smiseth OA, Appleton CP, *et al.* Recommendations for the Evaluation of Left Ventricular Diastolic Function by Echocardiography: An Update from the American Society of Echocardiography and the European Association of Cardiovascular Imaging. *Eur Heart J Cardiovasc Imaging* 2016; **17**: 1321–60.

5 Kundu S, Aulchenko YS, van Duijn CM, Janssens ACJW. PredictABEL: an R package for the assessment of risk prediction models. *European Journal of Epidemiology* 2011; **26**: 261–4.

6 Kuhn M. Building Predictive Models in R Using the caret Package. *Journal of Statistical Software* 2008; **28**: 1–26.

7 Robin X, Turck N, Hainard A, *et al.* pROC: an open-source package for R and S+ to analyze and compare ROC curves. *BMC Bioinformatics* 2011; **12**: 77.

8 Sjoberg DD. dcurves: Decision Curve Analysis for Model Evaluation. https://CRAN.R-project.org/package=dcurves.

9 Reddy YNV, Carter RE, Obokata M, Redfield MM, Borlaug BA. A Simple, Evidence-Based Approach to Help Guide Diagnosis of Heart Failure With Preserved Ejection Fraction. *Circulation* 2018; **138**: 861–70.

10 Barandiarán Aizpurua A, Sanders‐van Wijk S, Brunner‐La Rocca H, *et al.* Validation of the HFA‐PEFF score for the diagnosis of heart failure with preserved ejection fraction. *Eur J Heart Fail* 2020; **22**: 413–21.

11 Sanders‐van Wijk S, Barandiarán Aizpurua A, Brunner‐La Rocca H, *et al.* The HFA‐PEFF and H _2_ FPEF scores largely disagree in classifying patients with suspected heart failure with preserved ejection fraction. *Eur J Heart Fail* 2021; **23**: 838–40.

12 Parcha V, Malla G, Kalra R, *et al.* Diagnostic and prognostic implications of heart failure with preserved ejection fraction scoring systems. *ESC Heart Failure* 2021; **8**: 2089–102.

13 Ouwerkerk W, Tromp J, Jin X, *et al.* Heart failure with preserved ejection fraction diagnostic scores in an Asian population. *Eur J Heart Fail* 2020; **22**: 1737–9.

14 Obokata M, Kane GC, Reddy YNV, Olson TP, Melenovsky V, Borlaug BA. Role of Diastolic Stress Testing in the Evaluation for Heart Failure With Preserved Ejection Fraction: A Simultaneous Invasive-Echocardiographic Study. *Circulation* 2017; **135**: 825–38.

15 Andersen OS, Smiseth OA, Dokainish H, *et al.* Estimating Left Ventricular Filling Pressure by Echocardiography. *Journal of the American College of Cardiology* 2017; **69**: 1937–48.

16 Dal Canto E, Remmelzwaal S, van Ballegooijen AJ, *et al.* Diagnostic value of echocardiographic markers for diastolic dysfunction and heart failure with preserved ejection fraction. *Heart Fail Rev* 2022; **27**: 207–18.

17 Lancellotti P, Galderisi M, Edvardsen T, *et al.* Echo-Doppler estimation of left ventricular filling pressure: results of the multicentre EACVI Euro-Filling study. *European Heart Journal - Cardiovascular Imaging* 2017; **18**: 961–8.

18 Pak M, Kitai T, Kobori A, *et al.* Diagnostic Accuracy of an Integrated Echocardiographic Algorithm to Estimate Left Ventricular Filling Pressure. *JACC: Cardiovascular Imaging* 2022; published online June 15. DOI:10.1016/j.jcmg.2022.03.022.

19 van de Bovenkamp AA, Enait V, de Man FS, *et al.* Validation of the 2016 ASE/EACVI Guideline for Diastolic Dysfunction in Patients With Unexplained Dyspnea and a Preserved Left Ventricular Ejection Fraction. *JAHA* 2021; **10**: e021165.

20 Nikorowitsch J, Bei der Kellen R, Kirchhof P, *et al.* Applying the ESC 2016, H _2_ FPEF, and HFA‐PEFF diagnostic algorithms for heart failure with preserved ejection fraction to the general population. *ESC Heart Failure* 2021; **8**: 3603–12.

21 van de Bovenkamp AA, Wijkstra N, Oosterveer FPT, *et al.* The Value of Passive Leg Raise During Right Heart Catheterization in Diagnosing Heart Failure With Preserved Ejection Fraction. *Circ: Heart Failure* 2022; **15**. DOI:10.1161/CIRCHEARTFAILURE.121.008935.

22 Verbrugge FH, Reddy YNV, Sorimachi H, Omote K, Carter RE, Borlaug BA. Diagnostic scores predict morbidity and mortality in patients hospitalized for heart failure with preserved ejection fraction. *European J of Heart Fail* 2021; **23**: 954–63.

23 Nielsen OW, Valeur N, Sajadieh A, Fabricius-Bjerre A, Carlsen CM, Kober L. Echocardiographic subtypes of heart failure in consecutive hospitalised patients with dyspnoea. *Open Heart* 2019; **6**: e000928.

24 Pieske B, Tschöpe C, de Boer RA, *et al.* How to diagnose heart failure with preserved ejection fraction: the HFA–PEFF diagnostic algorithm: a consensus recommendation from the Heart Failure Association (HFA) of the European Society of Cardiology (ESC). *European Heart Journal* 2019; **40**: 3297–317.

25 Anker SD, Butler J, Filippatos G, *et al.* Empagliflozin in Heart Failure with a Preserved Ejection Fraction. *N Engl J Med* 2021; **385**: 1451–61.

26 Solomon SD, McMurray JJV, Anand IS, *et al.* Angiotensin–Neprilysin Inhibition in Heart Failure with Preserved Ejection Fraction. *N Engl J Med* 2019; **381**: 1609–20.

27 Pitt B, Pfeffer MA, Assmann SF, *et al.* Spironolactone for Heart Failure with Preserved Ejection Fraction. *N Engl J Med* 2014; **370**: 1383–92.

28 Massie BM, Carson PE, McMurray JJ, *et al.* Irbesartan in Patients with Heart Failure and Preserved Ejection Fraction. *N Engl J Med* 2008; **359**: 2456–67.

29 Yusuf S, Pfeffer MA, Swedberg K, *et al.* Effects of candesartan in patients with chronic heart failure and preserved left-ventricular ejection fraction: the CHARM-Preserved Trial. *The Lancet* 2003; **362**: 777–81.

30 Cleland JGF. The perindopril in elderly people with chronic heart failure (PEP-CHF) study. *European Heart Journal* 2006; **27**: 2338–45.

31 Solomon SD, Vaduganathan M, Claggett BL, *et al.* Baseline Characteristics of Patients With HF With Mildly Reduced and Preserved Ejection Fraction. *JACC: Heart Failure* 2022; **10**: 184–97.

32 Anker SD, Butler J, Filippatos G, *et al.* Baseline characteristics of patients with heart failure with preserved ejection fraction in the EMPEROR‐Preserved trial. *Eur J Heart Fail* 2020; **22**: 2383–92.

33 Sengupta PP, Shrestha S, Berthon B, *et al.* Proposed Requirements for Cardiovascular Imaging-Related Machine Learning Evaluation (PRIME): A Checklist: Reviewed by the American College of Cardiology Healthcare Innovation Council. *JACC: Cardiovascular Imaging* 2020; **13**: 2017–35.
